# New spinosaurids from the Wessex Formation (Early Cretaceous, UK) and the European origins of Spinosauridae

**DOI:** 10.1038/s41598-021-97870-8

**Published:** 2021-09-29

**Authors:** Chris T. Barker, David W. E. Hone, Darren Naish, Andrea Cau, Jeremy A. F. Lockwood, Brian Foster, Claire E. Clarkin, Philipp Schneider, Neil J. Gostling

**Affiliations:** 1 Institute for Life Sciences, University of Southampton, University Road, Southampton, SO17 1BJ UK; 2 Faculty of Engineering and Physical Sciences, University of Southampton, University Road, Southampton, SO17 1BJ UK; 3 School of Biological and Behavioural Sciences, Queen Mary University of London, Mile End Road, London, E1 4NS UK; 4 School of Biological Sciences, Faculty of Environment and Life Sciences, University of Southampton, University Road, Southampton, SO17 1BJ UK; 5 43125 Parma, Italy; 6 School of Environment, Geography and Geosciences, University of Portsmouth, Burnaby Road, Portsmouth, PO1 3QL UK; 7 Department of Earth Sciences, Natural History Museum, Cromwell Road, London, SW7 5BD UK; 8 Hessle, HU13 0HD UK; 9 Bioengineering Science Research Group, Faculty of Engineering and Physical Sciences, University of Southampton, Southampton, UK; 10 High-Performance Vision Systems, Center for Vision, Automation and Control, AIT Austrian Institute of Technology, Vienna, Austria

**Keywords:** Evolution, Palaeontology, Phylogenetics

## Abstract

Spinosaurids are among the most distinctive and yet poorly-known of large-bodied theropod dinosaurs, a situation exacerbated by their mostly fragmentary fossil record and competing views regarding their palaeobiology. Here, we report two new Early Cretaceous spinosaurid specimens from the Wessex Formation (Barremian) of the Isle of Wight. Large-scale phylogenetic analyses using parsimony and Bayesian techniques recover the pair in a new clade within Baryonychinae that also includes the hypodigm of the African spinosaurid *Suchomimus*. Both specimens represent distinct and novel taxa, herein named *Ceratosuchops inferodios* gen. et sp. nov. and *Riparovenator milnerae* gen. et sp. nov. A palaeogeographic reconstruction suggests a European origin for Spinosauridae, with at least two dispersal events into Africa. These new finds provide welcome information on poorly sampled areas of spinosaurid anatomy, suggest that sympatry was present and potentially common in baryonychines and spinosaurids as a whole, and contribute to updated palaeobiogeographic reconstructions for the clade.

## Introduction

Spinosaurids are among the most distinctive, unusual and controversial of theropods; they are characterised by an elongate, laterally compressed rostrum, sub-conical dentition and, in a subset of taxa, a dorsal sail formed by elongate neural spines. Their unusual cranial (and in derived forms, postcranial) morphology is atypical of non-avian theropods, and multiple lines of evidence point to an ability to exploit semi-aquatic niches^1–8^. A “generalist” or varied diet may have included a range of terrestrial and aquatic prey^9–12^ and was potentially influenced by individual size^13^ or habitat^14^. It has been suggested that spinosaurids became increasingly aquatic during their evolution^15^ and that highly modified taxa like *Spinosaurus* engaged in specialised underwater pursuit predation^7,8^. However, the sequence by which semiaquatic adaptations were acquired, and the degree of specialisation to aquatic life in *Spinosaurus* and other spinosaurids, remain debatable and the topic of ongoing research^16–19^.

The majority of spinosaurid material comes from Early and “mid” Cretaceous strata, although isolated dental remains suggest persistence of the group into the Late Cretaceous (Santonian)^20^. Views on how spinosaurids relate to other theropods, and on the relationships within Spinosauridae itself, are controversial. The majority of recent studies find spinosaurids to be nested within Megalosauroidea^21–23^, a position requiring an origin during the Early Jurassic at least. An alternative is that they are outside a Megalosauridae + Allosauroidea clade^24^. Spinosauridae itself is agreed by most to consist of the two clades Baryonychinae and Spinosaurinae^15,21,24–26^, although the number of valid taxa within these clades remains uncertain. Several—typically based on dental or fragmentary material—have been considered *nomina dubia* or subsumed into synonymy by some workers (e.g.^25,27^). Support for the spinosaurine/baryonychine dichotomy may not, however, be as strong as conventionally supposed^22^ and baryonychines may be paraphyletic to a monophyletic Spinosaurinae^28^.

Discussions of spinosaurid phylogeny have frequently been coupled with evaluations of the clade’s biogeographical history, in part because spinosaurines exhibit a strong Gondwanan signal and baryonychines a mostly Laurasian one (though neither clade is restricted to these two regions). However, rigorous attempts to reconstruct the clade’s palaeogeographic patterns have yet to be undertaken, largely due to the phylogenetic instability of spinosaurid taxa . Sereno et al.^25^ proposed an ancestral pan-Pangaean distribution followed by a Laurasian baryonychine and Gondwanan spinosaurine divergence driven by the opening of the Tethys. More recent discoveries (including of the Iberian *Vallibonavenatrix*, initially identified as a spinosaurine) provide complications for this model, rendering the biogeographical history of the clade unresolved^23^.

In the UK, spinosaurid fossils are restricted to the Lower Cretaceous Wealden Supergroup (see “Geological Context” below), a fossiliferous succession of mudstones, sandstones and siltstones well known for its dinosaurs and other vertebrates that is mostly exposed in the English south-east and Isle of Wight^29,30^. The partial holotype skeleton (NHMUK PV R9951) of *Baryonyx walkeri*—one of the world’s best spinosaurid specimens and the first to reveal the true appearance of members of this group—is from the Upper Weald Clay Formation (Barremian) of Surrey^1,31^. Its discovery precipitated the referral of various isolated Wealden Supergroup elements to this taxon; these include specimens from other Upper Weald Clay locations in Surrey, as well as the upper Berriasian–lower Valanginian Ashdown and Valanginian Wadhurst Clay formations of East Sussex^1,32^. Teeth referred to the *nomen dubium* “*Suchosaurus cultridens*”, from the Valanginian Grinstead Clay Formation of West Sussex, have also been attributed to *Baryonyx*^33,34^, although other work has favoured an indeterminate baryonychine position^30,35,36^.

Wealden Group spinosaurid remains have also been reported from the Isle of Wight, specifically from the Barremian Wessex Formation. Published material has, until now, consisted only of isolated teeth^37,38^ and the single dorsal vertebra IWCMS 2012.563^39^. Due to the temporal overlap of the Upper Weald Clay and Wessex Formations (both are Barremian^40^), these were previously assumed to belong to *Baryonyx* or a close relative; indeed, the teeth and vertebra have been referred to cf. *Baryonyx*/*Baryonyx* sp. and *Baryonyx* cf. *walkeri* respectively^30,38,41^. Attention has been drawn to differences in enamel ornamentation that exist between these isolated teeth and the teeth of the *B. walkeri* holotype, leading to suggestions that they might represent an additional baryonychine taxon^30,38,41^. The presence of multiple spinosaurids based on the presence of several tooth morphotypes has also been put forward for other palaeoecosystems (e.g.^42^). However, variation in spinosaurid crown ornamentation has uncertain taxonomic value within Spinosauridae^43^ and may be influenced by both tooth position and ontogeny^44,45^.

**Figure 1:**
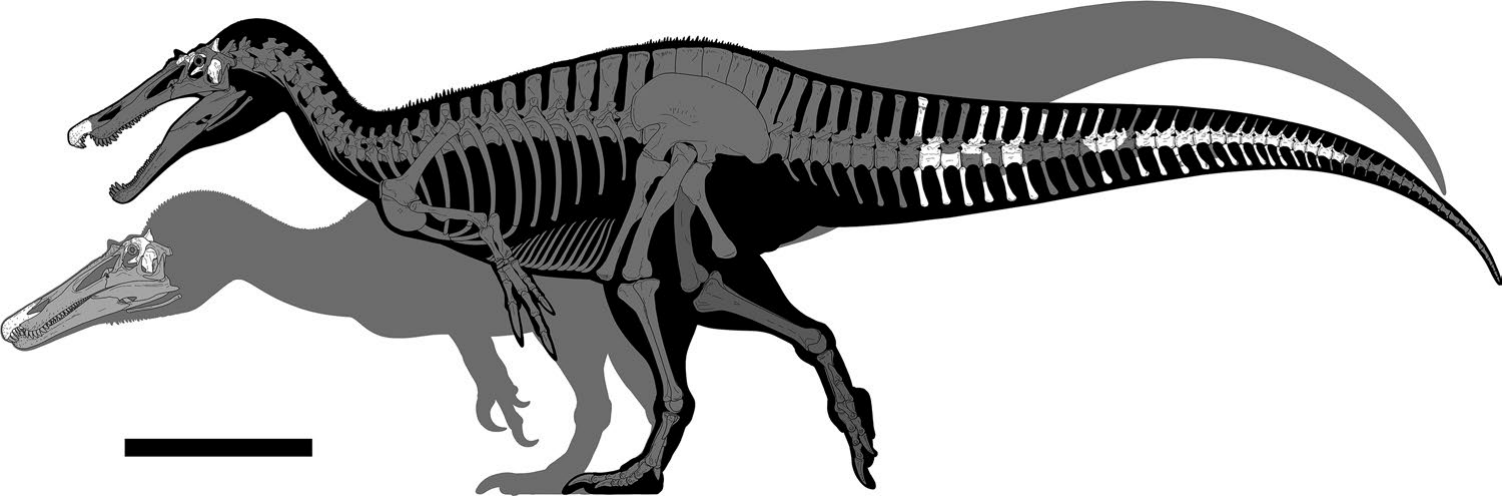
Known material referred to the baryonychines *Ceratosuchops inferodios* (rear) and *Riparovenator milnerae* (front) recovered at Chilton Chine (Isle of Wight, UK). White bones represent recovered elements. The arrangement of the elements in the caudal series is estimated; their relative position in the true series, and relationship with respect to each other (bar for those of the largely articulated mid-caudal series), are estimated. Image credit: Dan Folkes (CC-BY 4.0). Scale bar: 100 cm.

**Figure 2:**
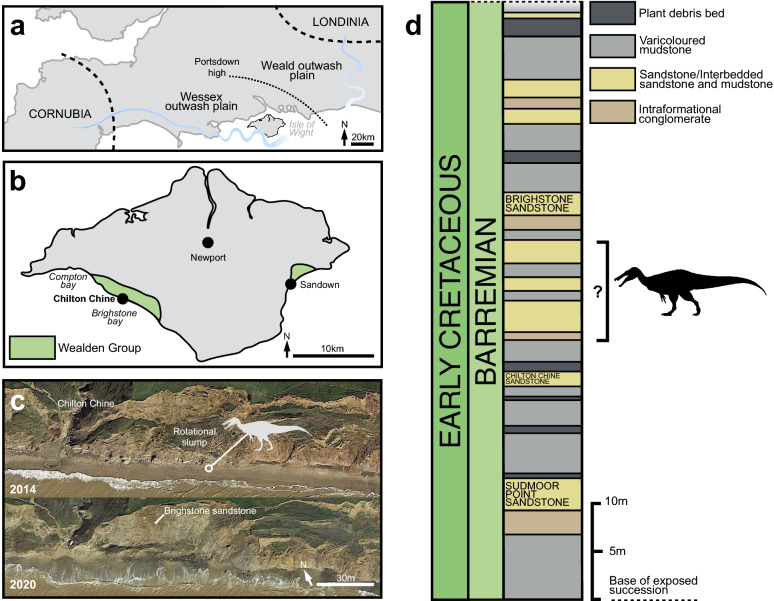
Locality information and stratigraphy of Chilton Chine. (**a**) Schematic palaeogeographic map of the Wessex and Weald sub-basins of southern England (modified from Penn et al., 2020); (**b**) map of the Isle Wight, highlighting the location of Chilton Chine and Wealden Group outcrops; (**c**) aerial photographs of Chilton Chine, highlighting the approximate position of the in situ material referred to *Riparovenator milnerae* (see SI) and the extensive coastal processes affecting the locality since the initial discoveries (map data: Google, Landsat/Copernicus, TerraMetrics, Maxmar Technologies); (**d**) schematic lithological log of the base of the exposed Wessex Fm. at Brighstone Bay (modified from Sweetman et al. 2014), highlighting approximate position of the *R. milnerae* in situ material. Silhouette credit: Dan Folkes (CC-BY 4.0).

The fragmentary and incomplete remains of two new baryonychine spinosaurid specimens were recovered at Chilton Chine on the Isle of Wight’s southwest coast and are herein named *Ceratosuchops inferodios* gen. et sp. nov. and *Riparovenator milnerae *gen. et sp. nov. (Figs. 1, 2). Both include partial skulls, the latter being associated with a series of caudal vertebrae (see supplementary information (SI) 1 for allocation of the material recovered and brief osteological descriptions). Surprisingly, both specimens differ from the broadly contemporaneous *B. walkeri* and from each other, and our interpretation demonstrates the presence of multiple spinosaurid taxa within the Wealden Supergroup. In this article, we explore their position within Spinosauridae via a new phylogenetic analysis and use this to re-evaluate spinosaurid palaeobiogeography. Finally, we discuss the possible implications of these new taxa for baryonychine diversity and ecology. A more detailed work on the osteology of the Wealden Group spinosaurids (including the additional spinosaurid elements recovered from Chilton Chine that could not be attributed to either taxon) is in preparation.

### Institutional abbreviations

IWCMS: Dinosaur Isle Museum (Isle of Wight County Museum Service), Sandown, UK; ML: Museu de Lourinhã, Lourinhã, Portugal; MNN: Musée National du Niger, Niamey, Republic of Niger; MSNM: Museo di Storia Naturale di Milano; NHMUK: Natural History Museum, London, UK.

## Geological context

The Lower Cretaceous Wealden Supergroup of southern England includes a succession of principally non-marine strata, accumulated between the late Berriasian and early Aptian. These are mainly deposited in two sub-basins: the larger Weald sub-basin of south-eastern England (where the strata are subdivided into the younger Weald Clay Group and older Hastings Group), and the smaller Wessex sub-basin of the Isle of Wight and central-southern England (composed of the younger Wealden Group and older Purbeck Limestone Group)^40^ (Fig. 2a). The Wealden Group on the Isle of Wight crops out along the southwest coast and less extensively so along the southeast coast (Fig. 2b), with both areas revealing exposures of the entirely Barremian Wessex Formation and overlying late Barremian-early Aptian Vectis Formation^46,47^. The Wessex Formation is formed of sandstones and varicoloured mudstones with interspersed plant debris beds. These sediments are mostly of alluvial origin and were deposited by a perennial, moderately sized, highly sinuous, west-to-east flowing river; some represent lacustrine environments as well^40,46,47^.

The new spinosaurid material reported here was collected at beach level, between 2013 and 2017, some of which originated from an exposure of the Wessex Formation located just east of Chilton Chine. The latter is a coastal geological feature situated approximately 1 km from Brighstone on the island’s southwest coast (Fig. 2b, c). The strata at Chilton Chine have experienced a substantial rotational slump, and additional, smaller slumps have further complicated the area’s stratigraphy. The braincase and caudal series referred to *Riparovenator* were recovered in situ and in close association (c. 10 m), likely from an unnamed layer between the Brighstone and Chilton Chine Sandstones (Fig. 2d); additional elements were recovered as isolated surface finds (see also SI). The sandstone matrix surrounding this specimen is largely fine-grained but does include small clasts; comparatively little plant debris (usually typical of the plant debris beds) was present during preparation. The *Ceratosuchops* premaxillae, braincase and referred postorbital were recovered from isolated sandstone blocks found on the foreshore, and as such their precise original stratigraphic location remains uncertain.

## Systematic palaeontology

- DINOSAURIA Owen, 1842.
- THEROPODA Marsh, 1881.
- TETANURAE Gauthier, 1986.
- SPINOSAURIDAE Stromer, 1915.
- BARYONYCHINAE Charig and Milner, 1986, sensu Sereno et al., 1998.
- CERATOSUCHOPSINI clade nov.
- LSID urn:lsid:zoobank.org:act:D2370EE2-B5B3-4921-B2F6-FA5207CE85BF.

*Definition:* The most inclusive branch-based clade containing *Ceratosuchops inferodios* but not *Baryonyx walkeri* and *Spinosaurus aegyptiacus*.

*Included taxa*: *Ceratosuchops inferodios; Riparovenator milnerae; Suchomimus tenerensis* Sereno et al., 1998.

*Diagnosis*: postorbital facet of frontal dorsoventrally thick (height more than 40% of length) and excavated by a deep, longitudinal slot; well-defined and strongly curved anterior margins of supratemporal fossa; occipital surface of the basisphenoid collateral oval scars excavated.

Genus *Ceratosuchops* nov.

LSID urn:lsid:zoobank.org:act:5EB49885-7AF9-45DF-854A-C75A1AED16A1.

*Etymology*: *kératos* (Greek, κέρας)—“horn”, prominent postorbital boss and rugose orbital brow; *soûkhos* (Greek, Σοῦχος)—“crocodile”; *óps* (Greek, ὄψ)—“face”.

*Type species: Ceratosuchops inferodios*.

*Diagnosis*: as for type and only species.

*Ceratosuchops inferodios* sp. nov.

LSID urn:lsid:zoobank.org:act:1957EEF7-F3DD-49FF-BB90-82F53EF8E34A.

*Etymology*: *īnfernus* (Latin)—underworld, hell; *erodiós* (Greek, ερωδιός)—heron, in reference to its presumed heron-like ecology.

**Figure 3:**
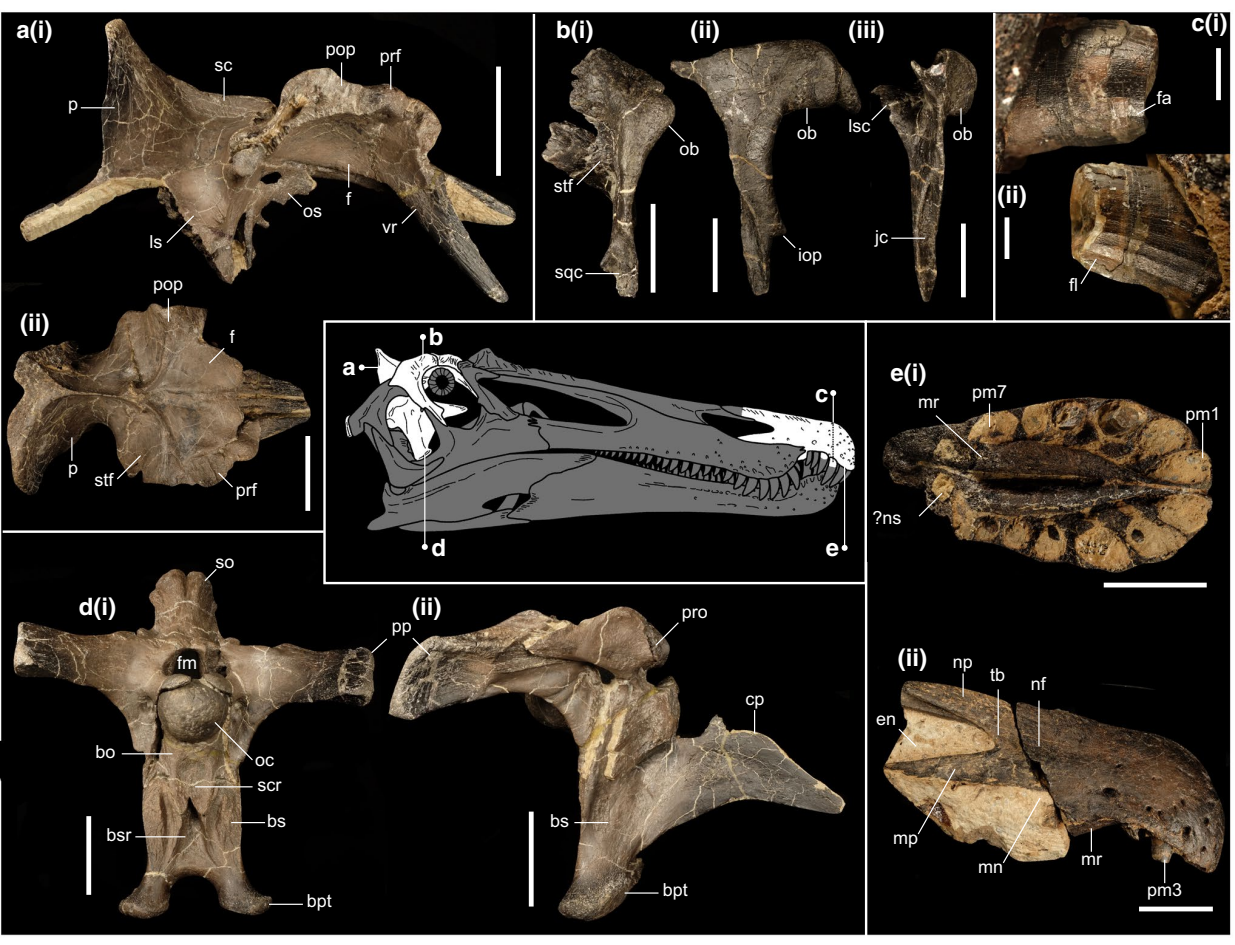
Cranial material of *Ceratosuchops inferodios*. (**a**) Holotype skull roof fragment (IWCMS 2014.95.1), in (i) right lateral and (ii) dorsal views; (**b**) referred right postorbital (IWCMS 2014.95.4), in (i) dorsal, (ii) lateral and (iii) posterior views; (**c**) close up of holotype in situ Rpm3 (IWCMS 2014.95.5) in (i) labial and (ii) lingual views; (**d**) holotype basicranium (IWCMS 2014.95.3), in (i) posterior (rearticulated with the supraoccipital + left otoccipital fragment IWCMS 2014.95.2) and (ii) right lateral views; (**e**) holotype premaxillae (IWCMS 2014.95.5, 2021.30), in (i) ventral and (ii) right lateral views. *bo* basioccipital, *bs* basisphenoid, *bpt* basipterygoid process, *bsr* basisphenoid recess, *cp* cultriform process, *en* external naris, *f* frontal, *fa* faceting, *fl* fluting, *fm* foramen magnum, *iop* infraorbital process, *jc* jugal contact, *ls* laterosphenoid, *lsc* laterosphenoid contact, *mn* maxillary notch, *mp* maxillary process, *mr* median ridge, *nf* narial fossa, *np* nasal process, *ns* nasal sinus, *ob* orbital boss, *oc* occipital condyle, *os* orbitosphenoid, *p* parietal, *pop* postorbital process, *pm(n)* premaxillary tooth/alveolus (tooth position), *prf* prefrontal, *pro* prootic, *sc* sagittal crest, *scr* subcondylar recess, *so* supraoccipital, *sqf* squamosal contact, *stf* supratemporal fossa, *tb* tuberosity, *vp* ventral process of the prefrontal. Skull reconstruction credit: Dan Folkes (CC-BY 4.0). Scale bars a–b, d–e: 50 mm; c: 5 mm.

*Holotype:* Associated premaxillary bodies (IWCMS 2014.95.5) and posterior premaxillary fragment (IWCMS 2021.30); a near complete but disarticulated braincase (IWCMS 2014.95.1-3) (Fig. 3).

*Referred material*: Right postorbital (IWCMS 2014.95.4) (Fig. 3).

*Diagnosis:* Baryonychine distinguished by the presence of the following unique traits: premaxillae displaying a pair of bilaterally located antenarial tuberosities; narrow (reversal of the ancestral megalosauroid condition) and ventrally restricted subcondylar recess of the basioccipital; oval scars of the basisphenoid excavated by deep, elongate sulci; anteroposteriorly thick interbasipterygoidal web; supraoccipital dorsal process possessing a gently curving posteroventral surface in coronal section.

This taxon can be further separated from other baryonychines by the following combination of traits: presence of narial fossae on the premaxilla (as in cf. *Suchomimus* but not *Baryonyx*); short subnarial (maxillary) process of the premaxilla (as in *Baryonyx* but not cf. *Suchomimus*); lack of premaxillary sagittal crest (as in *Baryonyx* but not cf. *Suchomimus*); curved anterior margin of the dorsal facet of the paroccipital process (angular in *Baryonyx* and probably *Riparovenator*); posterolaterally directed paroccipital processes of the otoccipitals (more laterally directed in *Baryonyx*); exoccipital components of the occipital condyle closely spaced (as in *Riparovenator* and cf. *Suchomimus* but not *Baryonyx*); subcondylar recess lacking mediolaterally thick lateral crests (as in cf. *Suchomimus* but not *Baryonyx* or *Riparovenator*); relatively stout supraoccipital dorsal process (as in *Baryonyx* but not cf. *Suchomimus*); lack of a dorsal extension of the basisphenoid recess under the basioccipital apron (recess extends dorsally in *Baryonyx* and *Riparovenator*).

*Type locality and type horizon*: Wessex Fm. (Barremian), Chilton Chine, near Brighstone (Isle of Wight, UK).

Genus *Riparovenator* gen. nov.

LSID urn:lsid:zoobank.org:act:9F4B6370-138E-443E-9650-DE6134FD9CC0.

Etymology: *Rīpārius* (Latin)–relating to the riverbank; *vēnātor* (Latin) –hunter.

*Type species: Riparovenator milnerae*.

*Diagnosis*: as for type and only species.

*Riparovenator milnerae* sp. nov.

LSID urn:lsid:zoobank.org:act:791F5DA4-1BDB-47DC-8ABF-BC24F14722B1.

*Etymology*: In honour of Angela Milner and her contributions to spinosaurid palaeobiology (and palaeontology as whole).

**Figure 4:**
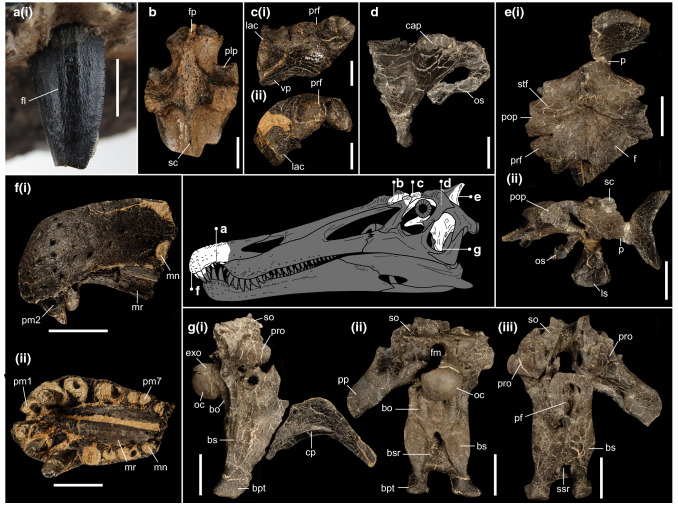
Cranial material of *Riparovenator milnerae*. (**a**) Close up of holotype in situ RpmVII (IWCMS 2014.95.6), in labial view; (**b**) referred posterior nasal fragment (IWCMS 2014.95.7) in dorsal view; (**c**) holotype left preorbital fragment (IWCMS 2014.96.3) in (i) lateral and (ii) anterodorsal view; (**d**) holotype right laterosphenoid (IWCMS 2014.96.2) in lateral view; (**e**) holotype skull roof and associated left laterosphenoid (IWCMS 2014.96.1) in (i) dorsal and (ii) left lateral views; (**f**) holotype premaxillary bodies (IWCMS 2014.95.6) in (i) left lateral and (ii) ventral views; (**g**) holotype basicranium (IWCMS 2020.448.1) in (i) right lateral (with fractured cultriform process IWCMS 2020.448.2), (ii) posterior and (ii) anterior views. *bo* basioccipital, *bs* basisphenoid, *bpt* basipterygoid process, *bsr* basisphenoid recess, *cap* capitate process of the laterosphenoid, *cp* cultriform process, *exo* exoccipital, *f* frontal, *fl* fluting, *fm* foramen magnum, *fp* frontal process, *lac* lacrimal, *ls* laterosphenoid, *mn* maxillary notch, *mr* median ridge, *oc* occipital condyle, *os* orbitosphenoid, *p* parietal, *plp* posterolateral process, *pop* postorbital process, *pm(n)* premaxillary tooth/alveolus (tooth position), *prf* prefrontal, *pro* prootic, *sc* sagittal crest, *scr* subcondylar recess, *so* supraoccipital, *ssr* subsellar recess, *stf* supratemporal fossa, *vp* ventral process of the prefrontal. Skull reconstruction credit: Dan Folkes (CC-BY 4.0). Scale bars (**a**): 5 mm; (**b**–**d**): 20 mm; (**e**–**g**): 50 mm.

*Holotype*: Associated premaxillary bodies (IWCMS 2014.95.6); a disarticulated braincase (IWCMS 2014.96.1, 2; 2020.448.1, 2); a left “preorbital” fragment (partial lacrimal and prefrontal) (IWCMS 2014.96.3) (Fig. 4).

**Figure 5:**
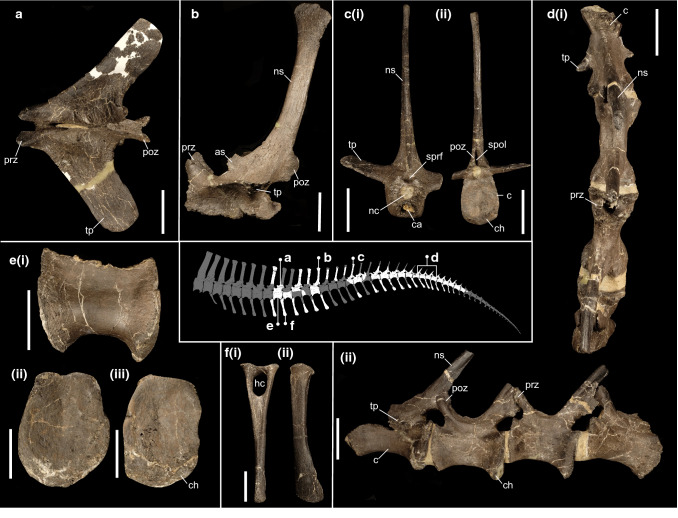
Caudal material referred to *Riparovenator milnerae*. (**a**) Anterior neural arch (IWCMS 2020.447.3) in dorsal view; (**b**) anterior neural arch (IWCMS 2020.447.2) in left lateral view; (**c**) partial middle vertebra (IWCMS 2020.447.8) in (i) anterior and (ii) posterior views; (**d**) articulated mid-caudal series (IWCMS 2020.447.12) in (i) dorsal and (ii) left lateral views; (**e**) anterior centrum (IWCMS 2020.447.5) in (i) left lateral, (ii) anterior and (iii) posterior views; (**f**) anterior chevron (IWCMS 2020.447.20) in (i) anterior and (ii) left lateral views. *as* anterior spur, *c* centrum, *ca* cavity, *ch* chevron contact, *h* haemal canal, *nc* neural canal, *ns* neural spine, *poz* postzygapophysis, *prz* prezygapophysis, *spof* spinopostygapophyseal fossa, *sprf* spinoprezygapophyseal fossa, *tp* transverse process. Tail reconstruction credit: Dan Folkes (CC-BY 4.0). Scale bars: 50 mm.

*Referred material.* A posterior nasal fragment (IWCMS 2014.95.7) (Fig. 4); an extensive caudal axial series (IWCMS 2020.447.1-39) (Fig. 5).

*Diagnosis:* Baryonychine distinguished by the presence of the following unique traits: notched dorsal orbital margin between prefrontal and postorbital process of the frontal; deeply inset facial nerve (CN VII) foramen that is largely obscured from lateral view; deep subcondylar recess (depth over 1/3 of its mediolateral width; depth less than 1/5 in other baryonychines); reduced exposure of ventral surface of the basipterygoid processes in lateral view.

This taxon can be further separated from other baryonychines by the following combination of traits: curved dorsal margin of the frontal process of the nasal in lateral view (margin effectively straight in *Baryonyx*); straight dorsal margin of the dorsum sellae (V-shaped in *Baryonyx* and *Ceratosuchops*); exoccipital components of the occipital condyle closely spaced (as in *Ceratosuchops* and cf. *Suchomimus* but not *Baryonyx*); mediolaterally thick crests bordering the subcondylar recess (as in *Baryonyx* but not *Ceratosuchops* or cf. *Suchomimus*); lateral margins of the basipterygoid processes concave in ventral view.

*Type locality and type horizon*: Between the Chilton Chine and Brighstone Sandstones, Wessex Fm. (Barremian), Chilton Chine, near Brighstone (Isle of Wight, UK).

## Results

### Parsimony analysis

**Figure 6:**
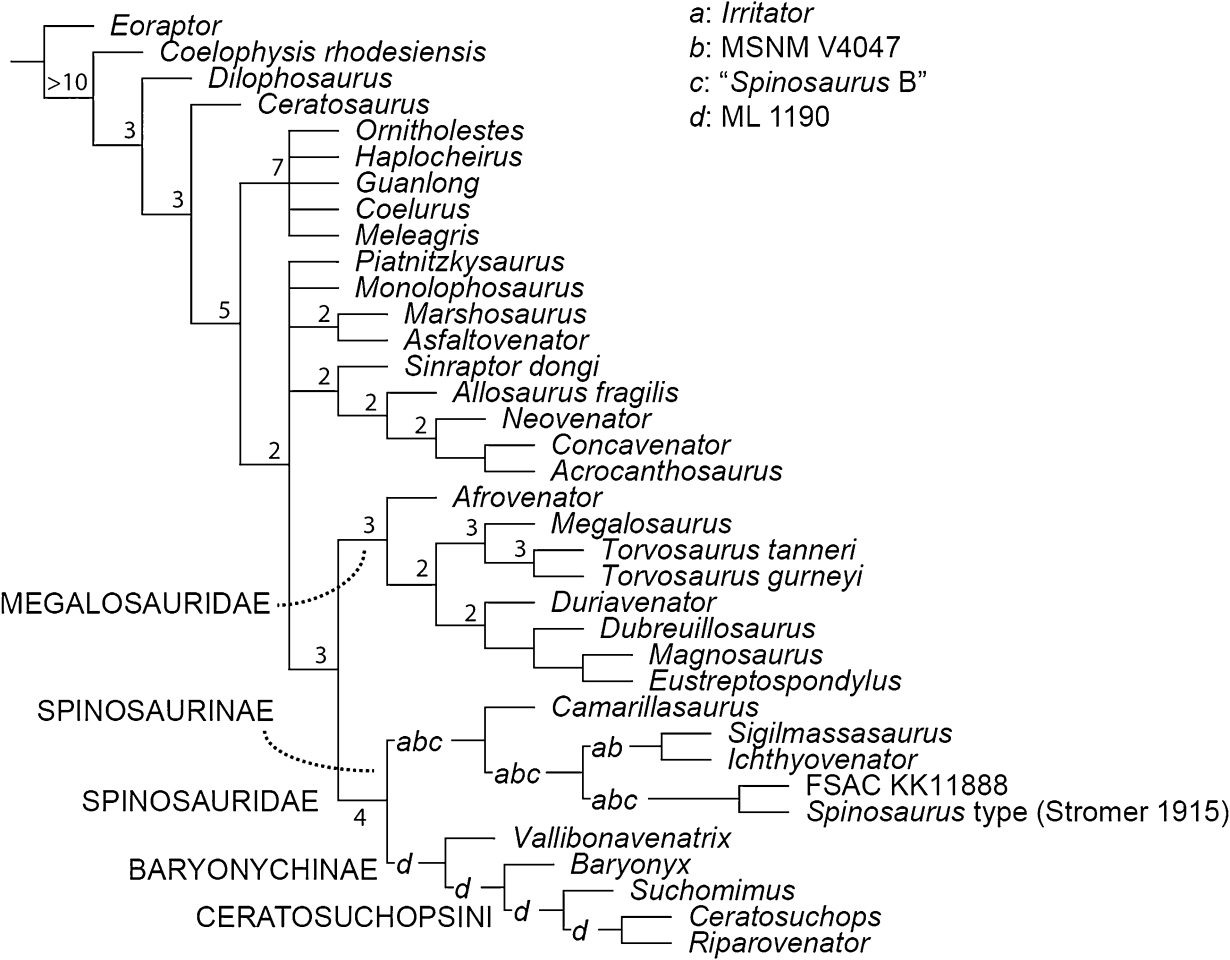
Phylogenetic relationships of Spinosauridae, based on parsimony analyses. Reduced consensus tree following a posteriori pruning of rogue spinosaurid OTUs. Values at nodes indicate the Bremer support values following pruning of rogue spinosaurid OTUs as well as select fragmentary taxa (see main text). Letters represent potential placement of rogue spinosaurid OTUs: a, *Irritator*; b, MSNM V4047; c, “*Spinosaurus* B”; d, ML 1190.

The parsimony analysis of 1810 characters and 40 Operational Taxonomic Units (OTUs) found 2660 shortest trees of 2448 steps each (CI = 0.4939; RI = 0.4551). The strict consensus is partially resolved, and we find support for the monophyly of Coelurosauria, Allosauroidea and Megalosauria. Among spinosaurids, the strict consensus topology weakly supports a dichotomous Baryonychinae-Spinosaurinae split, although their in-group relationships are completely unresolved. Pruning rogue spinosaurid OTUs (recovered by the TNT command *pcrprune*), improved in-group resolution but did little to alter the poor nodal (Bremer) support (Fig. 6). Jackknife resampling (see SI) also weakly supports the above-mentioned dichotomy, although it is unable to resolve the relationships between most of the spinosaurine in-group.

These results are in accordance with the majority of previous works regarding the well supported monophyly of Spinosauridae^11,21^ and the sister-group relationship between Baryonychinae and Spinosaurinae^15,21,24–26^ (the synapomorphies of these clades are listed in the SI). However, the results of the Templeton test (discussed below; see also SI) and poor nodal support reveal that the relationships of the spinosaurid in-group remain elusive and are hindered by the incompleteness of the sampled OTUs (see below). With respect to the broader affinities of Spinosauridae, our results generally conform to those of Cau^48^ and Rauhut and Pol^24^ in that they support the dissolution of the “traditional” avetheropodan node within Tetanurae, recovering instead an early-branching position for Coelurosauria, and (in this case) a polytomous Carnosauria that includes Allosauroidea and Megalosauroidea. In contrast, we find Megalosauridae to retain a traditional sister-group relationship with Spinosauridae.

### Bayesian analysis

**Figure 7:**
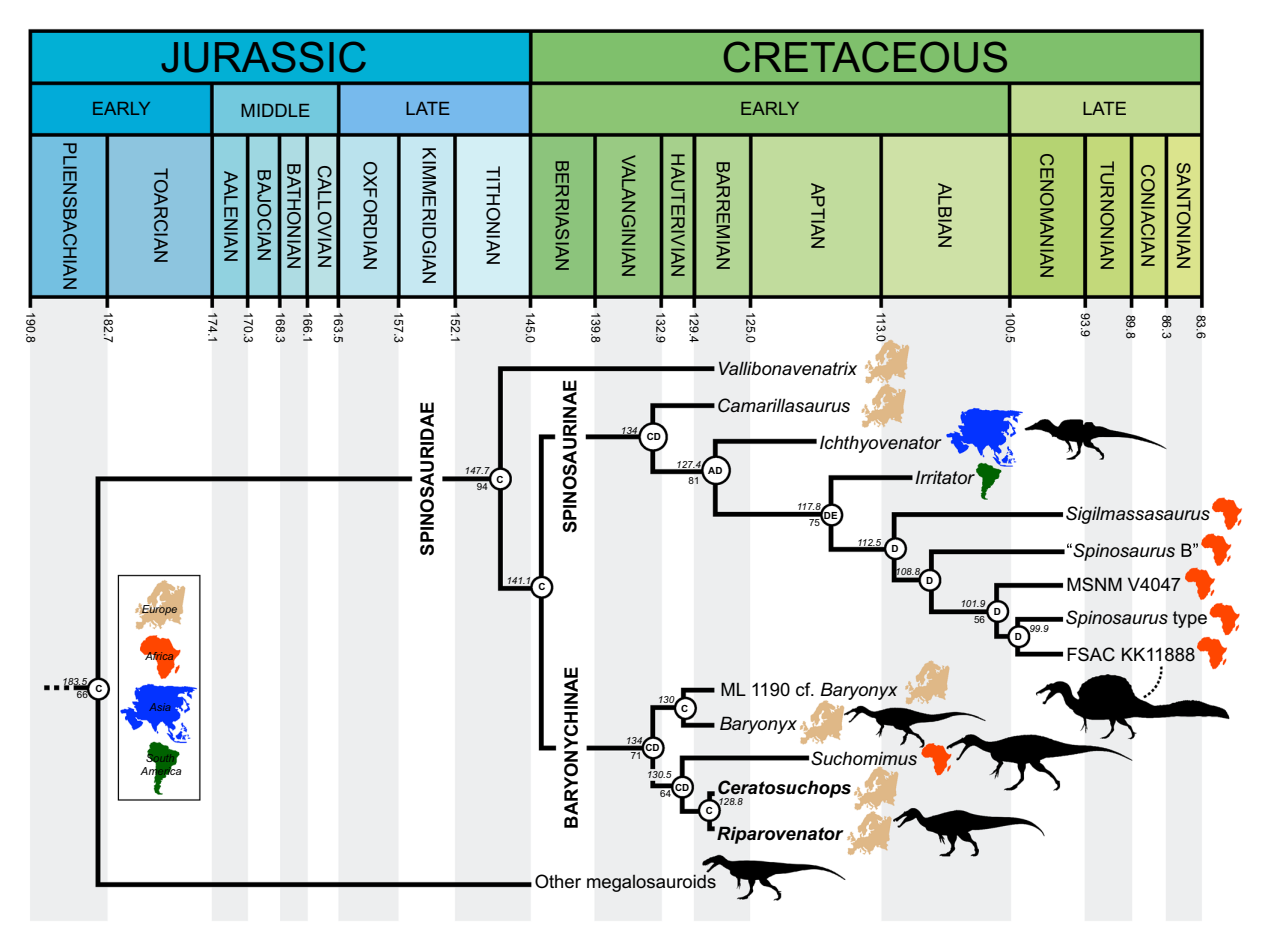
Time-calibrated phylogenetic relationships of Spinosauridae, based on the Maximum Clade Credibility Tree inferred by the Bayesian analysis (see SI for extended figure). Numbers at nodes represent node age (top, in million years) and posterior probability values > 50% (bottom). Letters at nodes refer to the most likely ancestral area reconstructed. Geologic timescale from Walker et al. (2018). *A* Asia, *B* North America, *C* Europe, *D* Africa, *E* South America. Silhouette credits: *Riparovenator*—Dan Folkes (CC-BY 4.0); *Baryonyx*, *Megalosaurus*, *Suchomimus*—Scott Hartman/Phylopic (CC-BY-NC-SA 3.0); FSAC KK 11,888—Scott Hartman; *Ichthyovenator*—Alex Vieira (CC-BY-NC-SA 4.0).

The Maximum Clade Credibility Tree (MCCT) obtained by the Bayesian inference analysis is in overall agreement with the strict consensus of the shortest trees recovered by the parsimony analysis, though resolution is substantially improved (Fig. 7, S1). The MCCT indicates that the [megalosaurid-spinosaurid] divergence occured at over 180 Mya and thus requires a c. 36 MY ghost lineage pre-dating an Early Cretaceous radiation of Spinosauridae. The monophyly of Spinosauridae is strongly supported (*pp* = 0.94), and relatively strong support is recovered for Baryonychinae (*pp* = 0.71) and Spinosaurinae (*pp* of the subclade excluding *Camarillasaurus* = 0.81). Within Baryonychinae, the two Wessex Fm. specimens are recovered as sister taxa and form a moderately supported clade with *Suchomimus* (*pp* = 0.64). Within Spinosaurinae, the hypothesis that all North African spinosaurines form a clade (to the exclusion of non-African taxa) is weak, with almost all in-group nodes possessing *pp* values < 50. The MCCT topology is thus equivocal on the distinction between *Sigilmassasaurus* and *Spinosaurus*. Of additional note is the early-branching position of *Vallibonavenatrix* outside the baryonychine-spinosaurine clade.

## Discussion

### Taxonomic interpretation

*Ceratosuchops inferodios* and *Riparovenator milnerae* represent new Wealden Group theropod taxa, differing from the other broadly contemporaneous spinosaurid *Baryonyx walkeri* in numerous anatomical features (see Diagnosis, Appendices 1 and 2).

Our taxonomic interpretation nevertheless overlaps with several vexing and interrelated issues: the relative maturity of the Wessex Formation taxa and the broadly coeval *Baryonyx* is unknown, as is the scope of variation amongst spinosaurid cranial characters. Regarding the former, no unambiguous methodology is currently available to distinguish the ontogenetic status of non-coelurosaurian theropods^49^: while similar fusion patterns (e.g. premaxillae, nasals) and relatively comparable body sizes suggest overarching similitudes between these Wealden Supergroup specimens, we accept that there are caveats to these indicators of maturity^49^. Multi-element histological sectioning of the more complete *Baryonyx* type specimen could nonetheless prove an informative starting point. We are cognisant that some of the differences we report for our two new baryonychine taxa are associated with individual and/or ontogenetic variation in better-sampled theropods (Appendix 1); indeed, intraspecific variation is seen to increase throughout the extreme ontogenetic series of the tyrannosaurid *Tyrannosaurus*^50^, for instance. Meanwhile, the presence of a mosaic of states in the Wessex Fm. pair, regarded as “mature” and “immature” in other theropods, could be interpreted as revealing high variation in the order of ontogenetic character maturation (sequence polymorphism) within a single taxon (as seen in non-tetanuran theropods^51^); conversely, it is unknown whether spinosaurids were affected by such variability and there are currently no good reasons to assume that all are applicable to the clade. Simply put, the interpretation of spinosaurid cranial characters requires larger samples before we can properly assess the tempo and sequence of ontogenetic character state maturation and scope of intraspecific variation. In the absence of these data, we opt to name the two Wessex Fm. taxa given the presence of unique characters and character combinations.

Osteologically, the Wessex Fm. spinosaurid pair provides welcome new cranial and caudal information, all of which will be expanded upon in future work. Of note is the rugose brow region of the skull in both specimens: in *Ceratosuchops*, this terminates posteriorly in an enlarged postorbital boss (Fig. 3b). Given the similarity in frontal postorbital facet shape in *Riparovenator* and cf. *Suchomimus* (MNN GDF 214), a similarly built postorbital (and thus brow region) is inferred. Such cranial ornamentation suggests a role in socio-sexual signalling^11^, although “overbuilt” brow regions may have also had a role in intraspecific antagonism^52^; biomechanical analyses, such as Finite Element analysis (FEA) could shed light on this interpretation. Several previously diagnostic cranial features in *Baryonyx*, such as the deep occiput (sensu^11^), are here interpreted as obsolescent given our recovered topologies and taxonomic interpretation of the new finds; others, for instance the cruciform process of the nasal (sensu^11^), require refinement to take into account differing morphologies. In the caudal series, the relatively elongate neural spines of *Riparovenator* in particular push back the appearance of a dorsoventrally deep tail in spinosaurids, this feature being previously known from the Aptian in *Ichthyovenator* and *Suchomimus*.

### Spinosaurid diversity in the Wealden Supergroup

Theropods appear rare in the Wealden Supergroup generally^30^, despite the strata being the subject of enthusiastic collecting efforts and producing numerous specimens of herbivorous dinosaurs^53^. As new Wealden Group taxa, *Ceratosuchops* and *Riparovenator* are thus substantial additions for the British theropod record, sharing the Wessex Fm. with at least three other mid-to-large theropods: the 7–8 m allosauroid *Neovenator*^53,54^, an indeterminate tetanuran^55^, and the 4–5 m tyrannosauroid *Eotyrannus*^56^. These new spinosaurid finds corroborate previous suggestions that Early Cretaceous Wealden Supergroup faunas were inhabited by more than one baryonychine taxon^30,38,41^, a discovery with potential implications for ecological separation within the clade, and for Spinosauridae as a whole.

The presence of more than one baryonychine taxon in the British Wealden was previously suggested from the discovery of at least two spinosaurid dental morphotypes^30,38,57^. However, it has been argued^27^ that the presence of multiple spinosaurid dental morphotypes within deposits containing a single known taxon was indicative of a “significant degree of subtle heterodonty” within the given putative taxon, rather than the presence of multiple taxa. Spinosaurids did exhibit heterodonty, but the discovery of these new Wessex Fm. baryonychines, coupled with the occurrence of at least two taxa and multiple dental morphotypes (representing baryonychines and spinosaurines) from the Early Cretaceous deposits of the Iberian Peninsula^58–62^, paints a more complex picture. Diagnostic Iberian taxa from the Barremian specifically include *Baryonyx*, *Vallibonavenatrix* and possibly *Camarillasaurus* (see below), though their remains currently occur in non-overlapping formations and the true taxonomic diversity of the Iberian sample remains elusive^61,62^. The validity of Iberian spinosaurine dental morphotypes has also been questioned^27^, despite there being some quantitative support for this referral (e.g.^63^) and the presence of unambiguous spinosaurine synapomorphies (carinae lacking serrations sensu Hendrickx et al.^64^). The presence of more than one spinosaurid taxon is also indicated by dental variation present in a Lower Cretaceous assemblage from Tunisia^65^. We are aware of, and reject, claims that the profound variation present in this sample—in this case, the presence of denticulated and non-denticulated morphotypes—can be explained by intraspecific variation^27^ in the absence of a clear morphological gradient or variably denticulated in situ dental series. Isolated Iberian spinosaurid teeth are known to possess variably denticulated carinae^62^; however their taxonomic implications are presently unclear.

Returning to the British Wealden Supergroup, labial and lingual enamel ornamentation (e.g. Fig. 3c) can be observed in both Wessex Formation taxa, as it can in isolated baryonychine teeth from this formation. This is a trait shared with “*Suchosaurus*” crowns from the neighbouring Weald sub-basin (and the Nigerien *Suchomimus*)^64^ and is distinct from the largely lingually fluted condition of *Baryonyx walkeri*^64^ (the caveat being the aforementioned variability and incomplete understanding of spinosaurid enamel ornamentation more generally). Regardless, assuming our taxonomic interpretation is correct, the discovery of the new Wessex Formation specimens renders the presence of *Baryonyx* in the Wessex Formation ambiguous. Indeed, the dorsal vertebra IWCMS 2012.563 previously referred to *Baryonyx*^30^ differs in its prominent spinodiapophyseal lamina and taller neural spine (potential damage to the *Baryonyx* type dorsal vertebrae notwithstanding)^30^. We argue that the specimen is best identified as an indeterminate baryonychine or spinosaurid; this can be extended to other isolated Wealden Supergroup spinosaurid material previously referred to “*Baryonyx*”.

Ecological demands require that large predators occur at low taxonomic diversity^66^. Despite this, numerous Jurassic and Cretaceous dinosaur assemblages include two or more comparably sized, morphologically similar, sympatric theropods^67^; examples are present in various geological formations worldwide (e.g.,^21,68–75^). The presence of two or more spinosaurid taxa in the same geological unit is therefore not without precedent and may in fact be typical^76^—the above mentioned Early Cretaceous of Iberia appears to testify this diversity. Indeed, when present, spinosaurids may have been locally abundant, perhaps when environmental circumstances benefited their specialised niche^11,20^, a feature potentially driving high diversity within the clade. As such, the possible presence of broadly coeval spinosaurid taxa in the British Wealden Supergroup may represent the norm based on our knowledge of other assemblages. A view popular in the Mesozoic dinosaur literature is that large theropod taxa can only coexist when anatomical traits indicative of resource partitioning are identifiable^77–80^. This view obviously has merit, but is not incompatible with the possibility that similar, closely related taxa can co-exist and even overlap in ecological requirements; niche separation may be temporal (seasonal or daily), spatial (between habitats within ecosystems), or conditional. It should also be noted that those baryonychines inhabiting the regions represented by the Wessex and Weald sub-basins may have been separated in habitat choice (possible sub-basin habitat differences or climates have been previously suggested^40,81,82^). Nonetheless, these hypotheses assume *Baryonyx*, *Ceratosuchops* and *Riparovenator* were contemporaries and subject to interspecific interactions. Alternatively, given the variable and intermittent sedimentation of fluvial systems^83^, the expanse of time deposited within the Upper Weald Clay and exposed Wessex Fms., and the difficulties correlating their respective stratigraphies, it remains possible one or more of these spinosaurids were separated by geological time.

### Phylogenetic analyses

We have determined through a large-scale phylogenetic analysis that the Wessex Formation specimens are more closely related to (a hypodigm OTU coding of) the Nigerien *Suchomimus tenerensis* than to *Baryonyx*, forming with it the newly recognised clade Ceratosuchopsini. This clade, and its internal relationships, is recovered under Bayesian and reduced consensus search strategies as well as through jackknife resampling (see SI), suggesting a potentially stable topology with albeit limited support. However, the extent to which the recovered topology is influenced by ontogenetically controlled character states is uncertain (see above). Additional characters, not currently included in the clade’s diagnosis, may further unite the Ceratosuchopsini, (e.g. shallow rise of the parietal nuchal crest; see Appendix 1); comparisons with sufficient, adequately preserved spinosaurid material would nevertheless be required.

Comparing the consensus trees produced by the two phylogenetic methodologies reveals grossly congruent topologies. However, some of the finer aspects of the ingroup relationships remain debatable: while Baryonychinae is recovered regardless of the phylogenetic methodology employed, we cannot reject alternative suboptimal scenarios where baryonychines form a paraphyletic grade to spinosaurines; constrained topologies forcing the Wessex Fm. pair outside of a *Baryonyx* + *Suchomimus* node, Ceratosuchopsini outside of a *Baryonyx* + Spinosaurinae node, and *Baryonyx* outside of a Ceratosuchopsini + Spinosaurinae node, required 2, 4 and 6 extra steps respectively, with all results insignificant under the Templeton test (see SI). This echoes previous works^22,28^ questioning the robusticity of the traditional spinosaurid in-group dichotomy. Similarly, differing topologies recovered within Spinosaurinae indicate a continued lack of in-group resolution. This impacts the perceived number of coeval North African spinosaurines, a topic that has attracted considerable debate in recent years^7,22,27,84^. The polytomous, parsimony-based strict consensus, coupled with the insignificance of the constrained analysis performed to group North African specimens (see SI), are unable to support or reject the existence of a spinosaurine subclade incorporating all North African material—a topology required to corroborate the proposed synonymy of these specimens (i.e. *Spinosaurus *sensu Ibrahim et al.^7^). This is somewhat echoed in our Bayesian analysis: a North African subclade is recovered, but its support is weak. Thus, the proposed synonymy of *Spinosaurus* and *Sigilmassasaurus*^27^ is regarded as equivocal here. It should be noted that these results might be affected by our decision to employ a composite OTU for *Sigilmassasaurus*, otherwise known from highly fragmentary type material; future analyses may consider removal of this OTU. Moreover, a better understanding of North African spinosaurine relationships requires the discovery of more complete individuals regardless.

Other minor topological differences include the Bayesian recovery of the Portuguese spinosaurid ML 1190 with the *Baryonyx walkeri* type specimen, a result differing from Arden et al.’s^15^ parsimony-driven placement of this specimen as an indeterminate spinosaurid. Our Bayesian findings are instead consistent with the specimen’s original referral to *Baryonyx*^36^. The low posterior probabilities for this grouping hinder support for this association, as does the instability of ML 1190 in our parsimony analysis, but these likely originate from the fragmentary nature of the latter OTU in particular. Meanwhile, *Vallibonavenatrix*—initially described as a spinosaurine^23^—is recovered as a baryonychine (Fig. 6) or a basal spinosaurid outside the baryonychine-spinosaurine spilt (Fig. 7), indicating an unstable position within Spinosauridae. Elsewhere, the recovery of *Camarillasaurus* as a spinosaurine (originally described as a ceratosaur^85^), lends further support to recently published reinterpretations of this taxon’s systematic position^61,76,86^. This interpretation may help explain the presence of spinosaurine-like teeth recovered from the same deposits^58^. The “wildcard” status of *Irritator* within Spinosaurinae in our parsimony-driven analysis is perhaps unexpected given its well-preserved cranial material. Whilst derived apomorphies in the dentition and narial region (that are absent in baryonychines) support its association to other spinosaurine OTUs with overlapping osteologies (e.g. MSNM V4047, *Spinosaurus* holotype), its unstable nature is likely explained by the inability to compare it to other taxa known predominantly from postcranial material.

Our phylogenetic analyses involved a substantial character list and large sample of spinosaurid OTUs, improving upon most previous studies that used comparatively smaller datasets. It also represents a first attempt to reconstruct spinosaurid palaeogeographic patterns using Bayesian methods. However, some spinosaurid OTUs could only be scored based on initial reports rather than first-hand observation or detailed monographs. This was the case for the *Suchomimus* OTU, for instance, and we favour using a hypodigm until the holotype specimen is thoroughly inventoried and described. We are nevertheless cognisant of the problematic inclusion of composite OTUs^87,88^. The lack of thorough descriptive works for many spinosaurid specimens remains a running hindrance in the study of the clade’s systematics and taxonomy, and is exacerbated by the incomplete nature of most specimens. This incompleteness likely contributes to the labile positions of various spinosaurid OTUs as well as the moderate to poor in-group node support throughout the consensus topologies.

### Spinosaurid palaeobiogeography

From a palaeogeographical perspective, our analysis supports a European origin of Spinosauridae generally consistent with the Laurasian origin model suggested by Milner^33^, with regionalisation and vicariance explaining the subsequent distribution of genus-level taxa. The Dispersal-Extinction-Cladogenesis (DEC) model supports the expansion of spinosaurid distribution during the first half of the Early Cretaceous to Asia and Western Gondwana, followed by progressive contraction of their distribution into the “mid” Cretaceous. In contrast, our findings are not consistent with Sereno et al.’s^25^ suggestion of an ancestral cosmopolitan distribution for spinosaurids followed by a vicariance-led divergence of Laurasian baryonychines and Gondwanan spinosaurines, and a *single* dispersal event invoked to explain the presence of *Suchomimus* in Africa. Our results instead indicate a European origin followed by at least two Early Cretaceous migrations to Africa, these leading, respectively, to *Suchomimus* and a clade within Spinosaurinae. An opposite direction of dispersal^89,90^ is not consistent with our results. This European origin and recovery of *Camarillasaurus* as an early-branching spinosaurine would appear to simplify the “complex” palaeogeographic pattern suggested by the presence of putative Iberian spinosaurines^23^. The Aptian extinction of the Eurasian Spinosauridae is potentially contradicted by evidence indicating their persistence well into the Late Cretaceous of Asia^20^. The highly fragmentary specimen (a single tooth) was not, however, included among our OTUs, and its omission has likely influenced the tempo of the clade’s perceived Eurasian extinction. Importantly, its potential baryonychine affinities may complicate the clade’s palaeogeographic patterns.

## Methods

### Phylogenetic analysis

Each of the new Wessex Formation specimens was entered into a phylogenetic dataset based on a modified version of the Cau^48^ analysis implemented by Dal Sasso et al.^91^, focusing on non-coelurosaurian tetanurans (see SI for further details). The Triassic saurischian *Eoraptor* was used as a root for the assessment of character polarity. The dataset was analysed using maximum parsimony as the tree search strategy using TNT version 1.5^92^. The search strategy involved 100 “New Technology” search analyses using the default setting, followed by a series of “Traditional Search” analyses exploring the tree islands found during the first round. Nodal support was calculated saving all trees up to 10 steps longer than the shortest topologies found and using the “Bremer Supports” function of TNT. In an attempt to improve tree resolution, rogue spinosaurid OTUs (*Irritator*, ML 1190, MSNM V4047 and “*Spinosaurus* B”) were identified using the command *pcrprune*^93^. Alternative relationships not recovered in the shortest found topologies were then enforced in TNT, and the statistical significance of their step difference from the most parsimonious trees found was calculated using the Templeton test^94^ implemented in TNT (see SI).

### Bayesian inference analysis

The phylogenetic dataset was analysed integrating the morphological data matrices with absolute ages of the least inclusive stratigraphic range including each terminal unit. The Sampled Ancestor Fossilized Birth Death Skyline Model (SAFBD;^95^) implemented in BEAST 2.4.4.^96,97^ was used as the tree model. In our analysis, rate variation across traits was modeled using the multi-gamma parameter (implemented for the analysis of morphological data in BEAST 2). The rate variation across branches was modeled using the relaxed log-normal clock model, with the number of discrete rate categories that approximate the rate distribution set as n − 1 (with n the number of branches), the mean clock rate using default setting, and not setting to normalize the average rate. Since the character matrix includes autapomorphies of the sampled taxa, the Lewis^98^ model was not conditioned to variable characters only. Stratigraphic information for the taxa was converted to geochronological ages. Stratigraphic data and age constraints for each terminal were obtained from the Paleobiology Database (http://paleobiodb.org/), checked against the International Chronostratigraphic Chart (http://stratigraphy.org/), and included as uniform priors for tip-dating. The extant taxon included (the avian *Meleagris*) calibrates the height for the tip-date setting (the uniform prior setting used for incorporating uncertainty in the age of the fossil taxa requires at least one terminal taxon to have the tip age fixed to a value, see^95^). The analysis used four replicate runs of 10 million generations, with sampling every 1000 generations, that were subsequently combined using LogCombiner 1.7.3 (included in the BEAST package)^96,97^. In the analyses, burnin was set at 20%. Convergence and effective sample sizes of every numerical parameter among the different analyses were identified using Tracer (included in the BEAST package). The root age of the tree model was conservatively set as a uniform prior spanning between the age of the oldest in-group taxa and 252 Mya (near the Permian–Triassic boundary), which consistently pre-dates the diversification of all theropod branches.

We used the MCCT resulting from the Bayesian analysis as a temporally calibrated phylogenetic framework for palaeobiogeographic reconstruction, inferring ancestral geographic placement of nodes using RASP (Reconstruct Ancestral State in Phylogenies)^99^. The distribution range of the taxa was a priori divided into five areas: Asia (A), North America (B), Europe (C), Africa (D), and South America (E). Each terminal taxon was scored for the geographic area character state according to the continent(s) it was recovered in. Biogeographic inferences on the phylogenetic frameworks were obtained by applying the Dispersal-Extinction-Cladogenesis model (DEC)^100^, with no a priori constraints on range and dispersal parameters.

## Supplementary Information

Supplementary Information 1.

Supplementary Information 2.

Supplementary Information 3.

Supplementary Information 4.

Supplementary Information 5.
