# Supplementary material for: New spinosaurids from the Wessex Formation (Early Cretaceous, UK) and the European origins of Spinosauridae
Source: Sci Rep. 2021 Sep 29;11:19340. doi: 10.1038/s41598-021-97870-8 (PMC8481559; doi:10.1038/s41598-021-97870-8)
Supplement: Supplementary file 1 — Supplementary Information 1. [file 41598_2021_97870_MOESM1_ESM.pdf]

SUPPLEMENTARY MATERIALS: New spinosaurids from the Wessex Formation (Early Cretaceous, UK) and the European Origins of Spinosauridae

Chris T. Barker[1,2]*, David W. E. Hone[3], Darren Naish[4], Andrea Cau[5], Jeremy A. F. Lockwood[6,7], Brian Foster[8],Claire E Clarkin[1,4], Philip Schenider[9,10], Neil J. Gostling[1,4]*

[1] Institute for Life Sciences, University of Southampton, University Road, Southampton, SO17 1BJ, UK

[2] Faculty of Engineering and Physical Sciences, University of Southampton, University Road, Southampton, SO17 1BJ, UK

[3]School of Biological and Behavioural Sciences, Queen Mary University of London, Mile End Road, London E1 4NS, UK

[4]School of Biological Sciences, Faculty of Environment and Life Sciences, University of Southampton, University Road, Southampton, SO17 1BJ, UK

[5]Independent, Parma, 43125, Italy

[6]School of Environment, Geography and Geosciences, University of Portsmouth, Burnaby Road, Portsmouth, PO1 3QL, UK

[7]Department of Earth Sciences, Natural History Museum, Cromwell Road, London SW7 5BD, UK

[8] Independent, Hessle, HU13 0HD, UK

[9]Bioengineering Science Research Group, Faculty of Engineering and Physical Sciences, University of Southampton, Southampton, UK

[10]High-Performance Vision Systems, Center for Vision, Automation & Control, AIT Austrian Institute of Technology, Vienna, Austria

Contents

1 Methods and Materials 3

1.1 Allocation of material recovered at Chilton Chine 3

1.2 Spinosaurid specimens used in the phylogenetic analyses 4

1.3 Brief comments on spinosaurid OTU selection and interpretation 5

1.4 Character amendments and additions 5

1.5 Synapomorphies of Spinosauridae 7

2 Templeton test results 9

3 Preliminary specimen descriptions 10

3.1 Select cranial measurements 10

3.2 *Ceratosuchops inferodios* 10

3.3 *Riparovenator milnerae* 12

4 Supplementary Figures 15

5 References 18

1 Methods and Materials

1.1 Allocation of material recovered at Chilton Chine

Here we provide brief comments regarding the allocation of the spinosaurid material recovered at Chilton Chine (Isle of Wight, UK) to the pair of individuals. In general, the material referred to *Riparovenator* is less well preserved than *Ceratosuchops*, possessing extensive cracking across the external surfaces.

Ceratosuchops inferodios

The material allocated to *Ceratosuchops inferodios* involves conjoined premaxillae, a braincase and a right postorbital, all found on the beach surface within separate blocks.

The premaxillae were found separately in two blocks on the beach surface; one contained the conjoined premaxillary bodies (IWCMS 2014.95.5) and the other its posterior processes (IWCMS 2021.30). These rearticulate well along the taphonomic crack that caused their separation, and are smaller than the rostral material recovered for *Riparovenator* (see below), supporting their association with the relatively smaller *Ceratosuchops* braincase. Further, their mode of preservation (which is superior to that of *Riparovenator*), suggests similar taphonomic histories. This confidence in the association permits the designation of the premaxillae to the holotype individual.

The disarticulated braincase material was recovered in very close association within a large but cracked sandstone block. This included a detached "skull roof complex" (paired frontoparietals, orbitosphenoids and laterosphenoids, with articulated right prefrontal) (IWCMS 2014.95.1), and a disarticulated supraoccipital+left otoccipital complex (IWCMS 2014.95.2) that had detached from the rest of the basicranium (articulated basioccipital-basisphenoid complex that includes both prootics and a disarticulated but closely associated right otoccipital) (IWCMS 2014.95.3). This unambiguously represents the remains of a single individual.

We refer an isolated right postorbital (IWCMS 2014.95.4) to the *Ceratosuchops* type individual as the dimensions of the articular facets of the postorbital, while damaged, match those of the respective frontal and laterosphenoid articulations. An adequate degree of articulation can be achieved and, in concert with the above comment regarding the mode of preservation, the element possesses a well-preserved external surface. Whilst uniformly darker relative to some of the material listed above, we note that several elements of the *Ceratosuchops* type specimen are variably coloured and possess darker regions (e.g. cultriform process, paroccipital processes, basipterygoid processes; see main text Fig. 3), suggesting the overall difference in colour may be the result of the local taphonomic processes affecting particular bone properties within a disarticulated skull.

Riparovenator milnerae

As mentioned in the main text, the *Riparovenator milnerae* material comprises of a mix of surface and in-situ finds. The former include associated premaxillary bodies (IWCMS 2014.95.6), a left preorbital fragment (IWCMS 2014.96.3) and a posterior nasal fragment (IWCMS.95.7). As above, the premaxillary bodies are consistent in size with the in-situ braincase elements attributed to *Riparovenator* (see below), and also share comparable modes of preservation; we thus designate it as part of the holotype. Similarly, the preorbital fragment articulates with the exposed facet on the holotype frontal and is clearly part of the same individual. We refer the nasal fragment to *Riparovenator* due to its size (the ventral frontal facet appears too large for the nasal process of the *Ceratosuchops* frontal) and similar state of preservation.

Additional cranial in-situ finds include the holotype braincase. This includes a skull roof (paired frontoparietals with articulated but incomplete right prefrontal and disarticulated left laterosphenoid and orbitosphenoid fragment) (IWCMS 2014.96.1) and an isolated right laterosphenoid (and partial orbitosphenoid) (IWCMS 2014.96.2), which were found on a separate occasion from the in situ basicranial complex (which included variably complete basioccipital, basisphenoid, supraoccipital, and paired prootics and otoccipitals) (IWCMS 2020.448.1, 2).

We also refer a series of in-situ axial elements (denoting at least 22 caudal vertebrae and include including four anterior centra, three anterior neural arches, and nine middle vertebrae and nine post-transition point vertebrae in largely good condition, as well as 21 chevrons and various neural spine fragments) (IWCMS 2020.447.1–39) to *Riparovenator* due to their close proximity (c. 10m) to the holotype basicranial complex.

1.2 Spinosaurid specimens used in the phylogenetic analyses

Spinosaurid OTU	Specimen(s)	Examined	Photo Credits	Literature
Baryonyx walkeri	NHMUK PV R9951	Yes (premaxillae, nasal, lacrimal, skull roof fragment, occiput)		[1,2]; axial series[3]; quadrate[4]; dentition[5].
cf. *Baryonyx walkeri*	ML1190	No		[6]
Camarillasaurus cirugedae	MPG-KPC1–46	No		[7-9]
Ichthyovenator laosensis	MDS BK10-01–25	No		[3,10]
Irritator challengeri (inc. *Angaturama limai*)	SMNS 58022, USP GP/2T-5	Photos of SMNS 58022	Marco Schade	Quadrate[4]; dentition[5]; [11,12].
Sigilmassasaurus brevicollis	BSPG 2006 I 53, 54, 55, 56; BSPG 2011 I 115, 116, 117, 118; BSPG 2013 I 95 CMN 41774, 41790, 41850, 41856, 41857, 41858; CMN 41857; MNN IGU11; NHMUK PV R 16427, 16434, 16435; P.P.No 481; CMN 50791; SGM–DIN 3, 5.	No		[3,13]
Spinosaurinae indet.	MSNM V4047	Yes		Dentition[5]; [14].
Spinosaurus aegyptiacus	BSP 1912 VIII 19	No		[3]; [15] (translation by Zanon, 1989); [16].
Spinosaurinae indet.	FSAC-KK 11888	No		[3,17,18]
"*Spinosaurus* B"	Nr. 1922 X 45	No		[3,19]
cf. *Suchomimus tenerensis*	MNN GDF 500–511, 214, MNN GAD 513, MNBH GAD 70. Additional dental interpretation based on comments in Hendrickx et al. (2019)	Photos MNN GDF 500-511, cast of MNN GDF 501, 3D model of MNN GDF 214	Serjoscha Evers, Marco Schade	Quadrate[4]; dentition [5]; [20]; furcula[21]; ulna [22]; anterior axial series[23]: Fig, 130.

Ceratosuchops inferodios (WightA)	IWCMS 2014.95.1–5, 2021.30	Yes		
Riparovenator milnerae (WightB)	IWCMS 2014.95.6–7, 2014.96.1–3, 2020.447.1–39, 2020.448.1–2	Yes		
Vallibonavenatrix cani	MSMCa-1, 3–5, 9–15, 18–20, 22–24, 27–28, 32–33, 53, 55	No		[24]

1.3 Brief comments on spinosaurid OTU selection and interpretation

The number of spinosaurine taxa represented by "mid-Cretaceous" remains from North Africa is controversial [3,18,23,25,26]. We use the name "*Spinosaurus aegyptiacus*" to refer specifically to the holotype material from Egypt [15]: other specimens which could eventually be referred to this taxon are referred to as "Spinosaurinae gen. et sp. indet." (e.g., the Moroccan specimens described by [14] and [17,18]). Stromer [15] described two cervical vertebrae for the *Spinosaurus* type specimen ("Wirbel a" and "b"); Smith et al., [16] tentatively identified "a" as an axis and "b" as a middle element, while Evers et al., [3] interpreted "a" as the third cervical 3 (i.e. anterior) and "b" as cervical 4 or 5 (i.e. middle); we follow the latter interpretation when scoring cervical characters. We use the name "*Sigilmassasaurus brevicollis*" [13] for the type material described by [13] but follow Evers et al., [3] in including additional material referred to this taxon. A theropod from the Bahariya Formation of Egypt described by Stromer [19] and informally referred as "*Spinosaurus* B" is here assumed to represent a single individual (see also [3,18]) and included in the analysis to test its affinities. As the precise inventory of material included in the *Suchomimus tenerensis* type specimen remains unclear (Sereno et al., [20] only mention a "partial disarticulated skeleton"), we treat this OTU as a hypodigm and also score referred material pending publication of thorough osteological descriptions of the spinosaurid material from Niger.

Cristatusaurus lapparenti, *Spinosaurus maroccanus* and *Oxalaia quilombensis* are based on fragmentary material [27-29]; since their character score strings are redundant with subsets of those of other included OTUs (e.g., *Oxalaia* is redundant with a subset of the scores of the unnamed Moroccan specimen MSNM V4704), we excluded them *a priori* from our analysis. We follow previous authors [2,11,20,27,30,31] in considering "*Angaturama*" a junior synonym of *Irritator*: both are scored into a single OTU (i.e., *Irritator*).

1.4 Character amendments and additions

The following character statements were emended:

- 974) Rephrased as: "Basioccipital contribution to foramen magnum margin: large, exoccipitals widely separated (0); reduced, exoccipitals closely placed (1)".
- 1120) Rephrased as: "Long bone, medullary cavity diameter: more (0); less (1) than 1/3 of bone diameter".

Four new characters replace previously included characters that are uninformative with the present taxon sample:

- 509): Basioccipital apron, basisphenoid overlap, dorsoventral extent: less (0); more (1) than 1/3 of the basisphenoid.
- 510): Basisphenoid, excavation of the collateral scars placed lateral to middle depression: absent (0); present and distinct on both sides (1).
- 511): Premaxilla, narial margin, overlap with oral margin: present (0); absent (1).
- 512): Premaxilla, interalveolar space between 3rd and 4th alveoli, mesiodistal diameter: less (0); subequal or more (1) than ½ of 4th alveolus mesiodistal diameter.

Additionally, Cau's [32] character list includes the following new character statements:

- 1782): Frontal, parietal suture, position relative to postorbital process: at same level (0); posteriorly placed, resulting in a concave posterolateral margin of frontal in dorsal view (1).

- 1783): Femur, shaft, distal half, elongate crest on posterior surface (accessory distal trochanter of Sereno et al. 1996): absent (0); present (1).
- 1784): Prootic, lateral surface, anterior end, large fossa housing foramina of cranial nerves V and VII: absent (0); present (1). (Brusatte et al., 2014).
- 1785): Anterior tympanic recess, development: narrow groove facing anterolaterally (0); wide foramen facing laterally (1). (Pei et al., 2017).
- 1786): Astragalus/tibiotarsus, distal condyles, ventral surface, shape in extensor/flexor view: flattened (0); rounded (1).
- 1787): Scapula, dorsal margin distal to acromion, flange distinct from acromion by cleft/concavity: absent (0); present (1).
- 1788): Manual digit IV: phalanx p2-IV: present (0); absent (1). Inapplicable in taxa lacking mc IV.
- 1789): Manual digit IV: phalanx p3-IV: present (0); absent (1). Inapplicable in taxa lacking mc IV.
- 1790): Manual digit V: phalanx p2-V: present (0); absent (1). Inapplicable in taxa lacking mc V.
- 1791): Dentary, first tooth crown, shape: similar to other rostral teeth (0); elongate, fang-like (1).
- 1792): †Dentary, lateral surface, neurovascular foramina, vertical sulci linking foramina to alveolar margin: absent (0); present (1).
- 1793): †Axis, centrum, shape in ventral view: mediolaterally constricted posterior to the intercentral suture (0); lateral margins subparallel (1). (Wang et al., 2017).
- 1794): †Presacral vertebrae, cervical and anterior dorsal centra, posterior surface, shape: flat to slightly concave (0); deeply concave (1). (Modified from Rauhut 2003; Holtz 2000; Carrano and Sampson 2008).
- 1795): Metatarsal IV, shaft, ventral view, proximodistally extended medial flange overlapping metatarsal III in articulated specimens: absent (0); present, metatarsal III shaft not exposed for most of its length ventrally (1).
- 1796): Premaxilla, lateral and dorsal surface, extensive pitting of neurovascular foramina: absent (0); present (1).
- 1797): Maxilla and dentary, paradental laminae (interdental plates), contact between adjacent elements: absent, widely spaced elements and crown base exposed lingually (0); contacting, crown base not exposed lingually (1).
- 1798): Pennaceous feathers (if present), vanes: open (no hocklets) (0); closed (hooklets present) (1).
- 1799): Elongate, semi-rigid tubular tegumentary structures (rachis or unbranched rachis-like elements, including those inserted on ulnar papillae): absent (0); present (1).
- 1800): Femur, intertrochanteric fossa between greater and anterior trochanter, nutrient foramen: absent (0); present (1). (Zanno et al. 2019).
- 1801): Postorbital, ventral (jugal) process, lateral surface, proximodistally (dorsoventrally) elongate ridge: absent (0); present (1). (Modified from Aranciaga et al. 2019).
- 1802): Prearticular, participation to milohioid foramen: absent (0); present as a distinct cleft or concavity (1). (Modified from Aranciaga et al. 2019).
- 1803): Ilium, preacetabular process, ventral margin, orientation of lateral surface relative to rest of blade: aligned dorsoventrally and facing laterally (0); flared laterally and facing ventrolaterally (1).
- 1804): Humerus, deltopectoral crest, anteromedial surface, pectoral scar along distal margin: absent or indistinct (0); present as a marked oval scar (1). (Rauhut et al., 2019).
- 1805): Basipterygoid processes, lateral view, angle formed with culfriform process and basal tubera (measured at basal tuber): less (0); more (1) than 30∞.
- 1806): Braincase, endocast, dorsal sinus (dural peak), distinction from the dorsal roof of the cast: poorly distinct (0); markedly distinct peak (1). (Paulina-Carabajal et al., 2019).
- 1807): Tibia, proximal end, posterior margin, accessory cleft lateral to main posterior sulcus between condyles: absent (0); present (1). (Modified from Ezcurra & Brusatte, 2011).
- 1808): Caudal vertebrae, accessory longitudinal ridge running along dorsolateral surface of neural arch, lateral to zygapophyses and medial to ribs: absent (0); present (1). (Rauhut, 2011).
- 1809): Caudal vertebrae, anterior and middle neural arches, posterior view, fossa on peduncle, ventrolateral to poszygapophyseal bases: absent (0); present (1). (Rauhut, 2011).

- 1810): Anterior caudal vertebrae, prespinal lamina, development: poorly- to moderately-developed, not markedly protruding anteriorly in lateral view (0); prominent, forming a distinct convex anterior margin of the neural spine at mid-height (1).

1.5 Synapomorphies of Spinosauridae

Common synapomorphies of the spinosaurid nodes reconstructed in all shortest trees found:

Spinosauridae:

Char. 11.1: premaxillae fused.
Char. 1796.1: external surface of premaxilla extensively pitted.
Char. 1485.1: ventral margin of premaxilla concave in lateral view.
Char. 14.1: fifth premaxillary alveolus present.
Char. 1717.1: sixth premaxillary alveolus present.
Char. 511.1: external naris retracted, does not overlap oral border of premaxilla.
Char. 582.1: mediolateral constriction of rostrum at premaxilla-maxilla transition.
Char. 1468.1: subnarial foramen replaced by a dorsoventrally oriented channel.
Char. 731.1: anteroventral border of dentigerous margin of maxilla curved dorsally.
Char. 18.0: medial palatal alae of maxilla expanded as large shelves.
Char. 1736.1: thick torus maxillaris confluent with anteromedial process.
Char. 707.1: anteromedial process of maxilla long, low and anteriorly projected beyond lateral surface of maxilla.
Char. 1175.1: promaxillary recess not exposed in lateral view.
Char. 1315.1: promaxillary recess penetrates anteroventral corner of antorbital fossa.
Char. 22.0: maxillary recess absent.
Char. 732.1: first maxillary alveolus oriented anteroventrally.
Char. 1500.1: nasal extensively overlaps nasal process of frontal.
Char. 662.1: angle between anterodorsal and ventral rami of lacrimal <60°.
Char. 756.0: straight anterior margin of lateral lamina of lacrimal.
Char. 1001.1: dorsoventrally vaulted frontals.
Char. 844.1: dorsoventrally elongate facial nerve foramen.
Char. 1751.1: medial condyle of quadrate with anterior concavity.
Char. 1747.0: quadrate ridge does not contact lateral condyle.
Char. 115.1: occipital condyle forms acute angle with basituberal processes in lateral view.
Char. 1192.1: basipterygoid processes placed ventral to basituberal region.
Char. 1566.1: deep ventral recess of basisphenoid.
Char. 758.1: anterodorsal margin of dentary raised relative to rest of dentigerous margin.
Char. 892.1: enlarged splenial foramen.
Char. 180.0: posterior margin of splenial straight (not concave).
Char. 1467.1: premaxillary and symphiseal dentary tooth crowns with apicobasal striations.
Char. 599.1: maxilla/dentary tooth crowns labiolingual diameter > 60% of mesiodistal diameter.
Char. 840.1: labial flutes on maxillary and post-symphiseal dentary tooth crowns.
Char. 1528.1: tooth roots narrowing basally.
Char. 200.1: distinct axial diapophyses.
Char. 1195.1: prominent axial parapophyses.
Char. 1550.1: large foramina on ventral surface of diapophyses in posterior cervical and cervicodorsal vertebrae.
Char. 943.1: pre- and postspinal laminae in dorsal vertebrae do not reach apex of neural spine.
Char. 555.1: anterior surface of cervical centra reniform, dorsally concave.
Char. 1194.1: pleurocoels penetrate parapophyses in middle cervical vertebrae.
Char. 1273.0: middle cervical neural spines subvertically oriented.
Char. 225.1: prominent ventral keel in anterior dorsal vertebrae.
Char. 230.1: anterior dorsal centra wider than tall in anterior view.
Char. 1740.1: dorsoventrally expanded anterior dorsal parapophyses.
Char. 1073.1: dorsal neural arches with spinodiapophyseal basal webbing.
Char. 250.1: neural spines in posterior dorsal, sacral and anterior caudal vertebrae at least 250% taller than long.
Char. 359.0: hyposphene absent in anterior caudal vertebrae.
Char. 562.0: anterior chevron lacking anteroproximal process.
Char. 406.1: distinct anterior convexity in the ambiens region of proximal pubis.

Char. 425.0: distal ischial shaft mediolaterally compressed.
Char. 693.1: distinct posterolateral projection on distal femur.

Baryonychinae:

Char. 1146.1: interdigitate premaxilla-maxilla suture in lateral view.
Char. 785.1: posterior end of nasal processes of premaxillae appressed.
Char. 72.1: dorsal surface of frontal with abrupt transition between anterior half and postorbital process.
Char. 78.1: parietal longer than ¾ of frontal.
Char. 509.1: basioccipital apron with extensive overlap over basisphenoid.
Char. 1151.1: marked muscle scars on basisphenoid.
Char. 1193.0: tooth serration in basal half of mesial carina.
Char. 212.0: anterior postaxial cervical neural spines not taller than long.
Char. 670.1: dorsal fossae in presacral neural arches.
Char. 660.1: accessory centrodiapophyseal lamina in dorsal vertebrae.
Char. 592.1: lateral margin of brevis fossa posterolaterally diverging in ventral view.
Char. 409.0: pubis lacking posterior extension of distal foot.

Spinosaurinae:

Char. 1136.1: maxilla included in narial fossa.
Char. 1050.1: dentigerous margin interrupted at the premaxilla-maxilla transition.
Char. 100.1: paraquadrate foramen reaches level of mandibular condyles.
Char. 1614.1: paired alveoli in premaxilla and anterior dentary.
Char. 153.1: maxilla/dentary paradental laminae indistinct.
Char. 154.1: maxillary/post-symphyseal tooth crowns unrecurved.
Char. 159.1: maxillary/post-symphyseal tooth crowns unserrated.
Char. 1507.1: maxillary/dentary tooth crowns with fluted labial surfaces.
Char. 1347.1: posterior dorsal neural spines taller than 12 times their width.
Char. 1349.0: absence of pneumatic foramina in anterior dorsal neural arches.
Char. 1608.1: reduced middle and posterior dorsal parapophyses.
Char. 1605.1: deep prezygocostal fossae in anterior caudal vertebrae.
Char. 358.1: centrocostal laminae in anterior caudal vertebrae.
Char. 626.1: prezygocostal laminae in anterior end middle caudal vertebrae.
Char. 929.1: elongate anterior and middle caudal ribs.
Char. 1034.1: mediolaterally thick middle caudal neural spines.
Char. 381.0: iliac supracetabular shelf reduced.
Char. 382.0: vertical crest dorsal to acetabulum absent.
Char. 245.1: tibia and femur relatively short compared to dorsal centra.
Char. 1120.1: reduced internal cavitation in long bones.
Char. 1069.1: collateral sulci in pedal unguals confluent with ventral surface of bone.
Char. 1433.1: pedal unguals with lateral ridges.

2 Templeton test results

Topology (T) 1: unconstrained MPT; T2: Wessex Fm. OTUs not included in the node (*Baryonyx*, *Suchomimus*); T3: Ceratosuchopsini not included in the node (*Baryonyx*, Spinosaurinae); T4: *Baryonyx* not included in the node (Ceratosuchopsini, Spinosaurinae); T5: *Spinosaurus sensu lato* (all North African Cenomanian OTUs forced as a monophyletic unit).

**Topology**	**Floater OTUs not constrained by enforced topology**	**Steps**	**Negative Ranks**	**Non-zero scores**	**Significance**	**Comment**
T1	None	2448				
T2	ML 1190, *Vallibonavenatrix*	2450	370	38	$p>0.05$	Non-significant
T3	ML 1190, *Vallibonavenatrix*	2452	430	42	$p>0.05$	Non-significant
T4	ML 1190, *Vallibonavenatrix*	2454	561	50	$p>0.05$	Non-significant
T5	None	2449	2	3	$p>0.05$	Non-significant

3 Preliminary specimen descriptions

Below are brief preliminary descriptions focusing on the major osteological features pending a more complete assessment (in prep.). Comparisons have been explored further in Appendix 1.

3.1 Select cranial measurements

Measurements (in millimetres) were taken using digital callipers and rounded to the nearest decimal point (Table X/Supp. Mat.). Asterisk denotes measurements influenced by taphonomic damage. AP= anteroposterior, DV=dorsoventral, ML=mediolateral). Values in square brackets indicate normalised ratio to occipital condyle width.

**Measurement**	***Ceratosuchops***	***Riparovenator***	***Baryonyx***
Premaxilla, maximum interpremaxillary (ML) width	86.3 [1.88]	100.6* [2.17]	83.3 [1.86]
Frontal, maximum interfrontal (ML) width	136.2 [2.97]	164 [3.53]	–
Frontal body (exc. nasal process), sagittal AP length	75.9 [1.66]	81.7 [1.76]	–
Parietal, sagittal AP length	81.6 [1.78]	93 [2.00]	–
Basioccipital, occipital condyle ML width	45.8	46.4	44.8
Basisphenoid, maximum ML width across oval scars	67.2 [1.47]	84.3 [1.82]	65.6 [1.46]
Supraoccipital, ML width across main body	111.6 [2.44]	112.6 [2.42]	104 [2.32]
Postorbital, maximum DV height	167.9	–	–
Postorbital maximum AP length	140.7	–	–
Postorbital, orbital boss DV height	48.7	–	–

3.2 *Ceratosuchops inferodios*

3.2.1 General notes

Taxon represented by a partial cranium (see above, main text). Elements generally well preserved. Cracking present across most surfaces but original bone texture remains largely visible. Premaxillary dentition poorly preserved with few teeth remaining in situ. Conjoined premaxillary nasal processes missing the posterior tip. Postorbital well preserved bar some damage to the ventral surface of the orbital brow and extremity of the ventral process. Braincase disarticulated into three closely associated parts. Abrasion to some articular surfaces and minor plastic deformation amongst certain braincase elements impairs clean manual rearticulation. Minor damage to the extremities of various braincase elements (e.g. left capitate process of laterosphenoid, ventral cristae tuberalis etc.) can also be observed. Comparative osteology is available in Appendix 1.

3.2.2 Osteology

Premaxillae

Premaxillae dorsally conjoined, interpremaxillary suture remains open anteriorly and ventrally. Body expanded to form a terminal rosette. Nasal and subnarial process project posteriorly and enclose an acute external naris. External surfaces peppered with many prominent neurovascular foramina. Dentigerous margin "hooked" anteriorly. Seven dental alveoli present per element; all located anterior to the external nares. No paradental laminae observed. Posteriorly keeled median ridges situated lingually to the alveoli and visible in lateral view. Two large vacuities extend anteriorly into the premaxillary body (made visible by transverse break), interpreted here as extensions of the nasal sinuses [18,33].

Dentition

Premaxillary dentition poorly preserved. Available teeth largely conidont with minor labiolingual compression. Size heterodonty noted along the tooth row, with largest crowns in the mesial three tooth positions. Fluted/facetted enamel observed on both the labial and lingual surfaces. Enamel texture appears veined (sensu Hendrickx, et al. [5]) where preserved intact.

Prefrontal

Prefrontal possessing a conspicuous, boss-like cornual process anteriorly. Anterior and ventral rami project from main body at an acute angle; lateral surface of latter possessing narrow facet for the lacrimal. Lateral surface posterior to rami excavated by a prominent notch.

Orbitosphenoids

Orbitosphenoids preserved as extremely thin elements whose sutural contacts with the adjacent laterosphenoids are not obviously discernible due to taphonomic cracking. Elements seemingly partaking in the olfactory (CN I), oculomotor (CN III) and the abducens (CN VI) nerve borders, while also enclosing the optic (CN II) nerve in its entirety.

Frontals

Frontal bodies coossified dorsally; however, interfrontal suture remains open ventrally. Nasal processes elongate and anteroventrally projecting; nasal facet does not overlap frontal body. Anterior frontal body concave in transverse section and bisected by a low, incomplete sagittal ridge. Anterior margins of the supratemporal fossae sharply defined and laterally curving. Prefrontal facet deepest anteriorly. Postorbital processes well developed and laterally projecting, forming posterior wall of a dorsally right-angled articular space for the prefrontal; anterior surface of process possesses articular facet to contact prefrontal. Frontals excluded from the orbital margin. Frontals convex in lateral view, exposing the orbital fossae. Frontoparietal suture V-shaped; suture marked medially by small bilaterally projecting frontoparietal processes.

Parietals

Parietals hourglass in shape in dorsal view. Sagittal crest thin and undivided; supratemporal fenestrae narrowly separated on skull roof. Posterior nuchal crest thin and projects laterally and somewhat dorsally; mid-dorsal margin flattened. Posterolateral ala of the parietal present as a narrow, spike-like process.

Laterosphenoids

Antotic crests bilaterally asymmetrical (right crest forms a sharp ridge terminating ventrally in a small protrusion; left poorly developed). Capitate process ovate and generally mediolaterally directed. Laterosphenoids form anterior margins of the trigeminal nerve (CN V) foramina; ophthalmic ramus (CN V_1) excavates ventral element via sulcus. Likely forming posterior borders to the oculomotor (CN III) and the abducens (CN VI) nerve foramina (but see orbitosphenoids above).

Prootics

Trigeminal nerve (CN V) foramen margin largely formed by prootic. Element also delineates anterior border of the fenestra ovalis and entirely encapsulates the facial (CN VII) nerve. Prootic overhangs a shallow columellar recess. Internal facial (CN VII) and vestibulocochlear (CN VIII) nerve foramina visible endocranially due to disarticulation of the braincase, as are the ventral portions of the floccular recesses. Prootics seemingly contribute to dorsal pituitary fossa; if correct, the abducens nerve (CN VI) foramina exit through prootics and open into fossa.

Otoccipital

Otoccipitals formed via the extensive fusion of the opisthotics and the exoccipitals. Both otoccipitals contribute extensively to the lateral margins of the foramen magnum, bar for minor median input by the supraoccipital dorsally and basioccipital ventrally. Paroccipital processes well developed and posterolaterally projecting, with concave anterolateral surfaces. Crista tuberalis forming the posterior borders of the fenestra ovalis, and encapsulating the adjacent fenestra pseudorotunda (glossopharyngeal nerve (CN IX) foramen). Posterior otoccipital pierced by three neurovascular foramina (for CN X–XII), lateral to the occipital condyle.

Supraoccipital

Supraoccipital producing a conspicuous dorsal process projecting from the platform-like main body. Posterior surface of the dorsal process excavated along its dorsal midline by a shallow sulcus; median crest or keel lacking. Posterior middle cerebral vein foramina penetrate the supraoccipital bilaterally at the base of the dorsal process, internal foramina also visible endocranially, as are the dorsal portions of the floccular recesses. Main body of the supraoccipital indented by bilateral crescentic depressions; depressions contour tab-like processes.

Basioccipital
Occipital condyle largely hemispherical and mainly formed by basioccipital. Condylar neck short and subtly inset from condyle margins. Basioccipital apron convex dorsally and indented ventrally by a subtle subcondylar recess; the recess excavates well below the occipital condyle. Basitubera unclear but presumably represented by the pair of flat ventral extensions of the apron that overlap the basisphenoid and form the apex of the basisphenoid recess margin.

Basisphenoid
Basisphenoid and parasphenoid indiscernibly fused together. Cultriform process mediolaterally thin and anteriorly projecting (when in life position), with a gently curved ventral margin. Process excavated dorsally by *sulcus septalis* (for the presumably cartilaginous interorbital septum) and ventrally by the anterior portion of the subsellar recess. Anterior (=lateral) tympanic recesses bilaterally excavate basisphenoid and are overhung by conspicuous preotic pendants. Oval "scars" (*sensu* Bakker, et al. [34]) well developed; lateral margins only incipiently curved. Basisphenoid recess opening triangular; recess anteroposteriorly deep but fails to extend dorsally under the basioccipital apron. "Scars" deeply excavated by narrow, dorsoventrally trending sulci. Basipterygoid processes strongly taper anteriorly and generally rounded posteriorly however asymmetry may be observed in ventral view. Both project anteroventrally and slightly laterally (in life position). Interbasipterygoidal space largely semicircular. Interbasipterygoidal web anteroposteriorly thick.

Postorbital
Postorbital brow displaying a large, laterally projecting ovoid boss at the posterodorsal corner of the orbit. Anterior dorsal surface excavated by supratemporal fossa. Ventral process elongate and ventrally tapering, with a U-shaped cross section at its midshaft due to concave posterior jugal facet; jugal facet visible in lateral view. Infraorbital process present as a small, triangular projection.

3.3 *Riparovenator milnerae*

3.3.1 General notes
Taxon represented by a partial cranium and semi-articulated portion of the caudal vertebral series (see above, main text). Taphonomic damage is more extensive relative to *Ceratosuchops*, with substantial cracking evident across the cranial elements in particular. Various element extremities lost (e.g. premaxillary processes, right paroccipital process, supraoccipital dorsal process, various caudal transverse processes and neural spines) or damaged (e.g. cultriform process broken from the basisphenoid, crushed caudal centra). Plastic deformation mainly affecting a select few processes of the caudal series and not observed in the braincase. Interpremaxillary suture open and premaxillary bodies disarticulated due to damage sustained along presumably fused dorsal margin; few teeth remaining in situ and erupted teeth damaged. Braincase disarticulation includes the skull roof from the basicranium, bilateral isolation of the laterosphenoids, and detachment of the left preorbital elements and otoccipital. For comparative osteology, see Appendix 1.

3.3.2 Osteology

Premaxillae
Premaxillae only preserving the anterior terminal rosette. Dentigerous margin "hooked" anteriorly; seven dental alveoli present per element. Interdental plates not observed. Lateral surfaces peppered with many prominent neurovascular foramina. Keeled median ridges visible in lateral view.

Dentition
Size heterodonty noted along the tooth row. First premaxillary alveoli notably smaller relative to the second. Preserved teeth largely conidont with minor labiolingual compression. Teeth posteriorly recurved. Fine serrations (5-8 denticles/mm) noted on intact carinae. Fluted/facetted enamel observed on both the labial and lingual surfaces. Enamel texture appears veined on intact replacement teeth apices.

Nasals
Nasals coossified along their midline into a low sagittal crest that develops posteriorly into an abraded median knob. Nasal ornamentation cruciform in dorsal view. Posterolateral processes triangular and horizontally projecting. Frontal (=posterior) process stout with a curved dorsal margin. Small fragment of a ventral median ridge, visible on posterior ventral surface.

Lacrimals and prefrontals
Prefrontal possessing a boss-like cornual process. Anterior and ventral rami diverge at an acute angle; latter possesses facet for the lacrimal along lateral surface. Lateral surface posterior to the rami notched. Posterior lacrimal fragment articulated to the left prefrontal via a visible, foramina-lined suture.

Orbitosphenoids
Orbitosphenoid fragments preserved but offer little of osteological note.

Frontals
Frontal bodies coossified dorsally; however, interfrontal suture remains open ventrally. Incomplete nasal processes anteroventrally projecting; nasal facet does not overlap frontal body. Anterior frontal body concave in transverse section and bisected by a low, incomplete sagittal ridge. Anterior margins of the supratemporal fossae sharply defined and laterally curving. Frontals arched in lateral view to expose the orbital fossae. Prefrontal facet deepest anteriorly; posterior portion of facet accepts prefrontal via a transversely V-shaped facet. Postorbital processes well developed and lacking an anterior facet for prefrontal; the processes anterolateral corner is thus exposed, potentially allowing for a minor frontal contribution to the dorsal orbital margin. Frontoparietal suture initially transverse medially, before coursing posteriorly for a short distance and then regaining a generally transverse trend. Frontoparietal processes prominent but asymmetrical.

Parietals
Parietals hourglass-shaped in dorsal view. Sagittal crest present; supratemporal fenestrae narrowly separated on skull roof. Nuchal crest present and flattened along dorsal margin, but badly damaged more generally.

Laterosphenoids
Antotic crest poorly developed with rounded cross section, leading to an ovate and mediolaterally directed capitate process. Lateral (adductor) surface producing a low tuberosity posterior to antotic crest. Sulcus for the ophthalmic ramus of the trigeminal nerve present ventrally.

Prootics
Trigeminal nerve foramen margin principally formed by prootic. Element delineates anterior border of the fenestra ovalis and entirely encapsulates the facial (CN VII) nerve. Facial nerve (CN VII) deeply inset and obscured in lateral view. Posterior process overhangs columellar recess. Abducens nerve (CN VI) foramina open into pituitary fossa and are separated by a deep, posteriorly directed conical space.

Otoccipitals
Otoccipitals formed via the extensive fusion of the opisthotics and the exoccipitals. Contribution by elements to foramen magnum margin substantial, bar for minor median input by the supraoccipital dorsally and basioccipital ventrally. Paroccipital process well developed with concave lateral anterior surface. Posterior otoccipital pierced by three foramina lateral to the occipital condyle.

Supraoccipital
Dorsal process largely missing, although coronal section clearly demonstrates pinching of ventral posterior surface into a low, rounded midline ridge. Body indented with bilateral crescentic depressions. Posterior middle cerebral vein foramina penetrate the supraoccipital bilaterally at the base of the dorsal process.

Basioccipital
Basioccipital forming majority of hemispherical occipital condyle; condyle neck short and inset from condyle margins. Basioccipital apron extends ventrally and excavated by a deep subcondylar recess; recess extends from ventral neck of the condyle (but possibly exaggerated by taphonomic damage) and delimited laterally by robust crests. As above, basitubera presumably represented by the pair of flat ventral extensions of the basioccipital apron that overlap the basisphenoid and form the apex of the basisphenoid recess.

Basisphenoid
Cultriform process mediolaterally thin; tip ventrally downturned to give ventral margin a more strongly arched outline. Process excavated dorsally by *sulcus septalis* and ventrally by the anterior portion of the subsellar recess; subsellar recess bisected by thin lamina. Anterior (=lateral) tympanic recesses

bilaterally excavating lateral basisphenoid and overhung by conspicuous preotic pendants. Oval "scars" well developed with rounded lateral margins. Basisphenoid recess opening triangular; recess anteroposteriorly deep and extends dorsally under the basioccipital apron. "Scars" excavated by short, shallow depressions. Basipterygoid processes strongly tapers anteriorly; posterior margins somewhat pinched in ventral view. Exposure of ventral surface of basipterygoid process reduced in lateral view. Processes project anteroventrally and slightly laterally when in life position. Interbasipterygoidal space with somewhat flattened dorsal margin in posterior view. Interbasipterygoidal web relatively thin anteroposteriorly.

Caudal vertebrae
Partially articulated caudal series includes at least 22 vertebrae and 18 chevrons. Series preserves "transition point" between vertebrae possessing and lacking caudal ribs [35]; all bar nine of the recovered vertebrae are anterior to this point. Closure of neurocentral sutures follows a posterior-anterior trend.

Centra shallowly amphicoelous. Anterior centra are only slightly longer than tall and constricted about their mid point; centra become more elongate posteriorly. Centra internally hollow.

Neural arches typified by tall neural spines; spine height decreases posteriorly but remains taller than centra facet heights well into middle series where preserved. Intact anterior and middle neural spines show minor anteroposterior expansion at the dorsal extremities. Spine inclination increases posteriorly. Spine cross-section thin and anteroposteriorly elongate in anterior series, becoming ovate more posteriorly. Accessory neural spines (=anterior spurs) project from the anterior base of pre-transition point neural spines; reduced to small ridges in elements immediately posterior before disappearing. Webbing present at lateral spine base of anteriormost elements.

Prezygapophyses anterodorsally projecting in lateral view; anterior facet overlap minimal to absent. Prezygapophyseal facets face mainly medially. Spinoprezygapophyseal fossa narrow and deep anteriorly, becoming boarder and shallower in middle elements. Postzygapophyses thin and medially trending in more anterior elements, becoming more parallel posteriorly.

Caudal ribs generally project posterolaterally with minor dorsal inclination in undeformed anterior elements; reduced to small "fin"-like process by transition point. Ventral laminae absent bar for stout buttress (?centrodiapophyseal lamina) in anterior neural arches.

Chevrons lacking anterior processes. Haemal canal generally tall and ovate in anterior elements, becoming stouter and flat-topped posteriorly. Chevron articular facets divided into anterior and posterior portions. Haemal spines elongate with anteroposteriorly spatulate ventral tips in anterior elements; spine rod-like in posterior elements.

4 Supplementary Figures

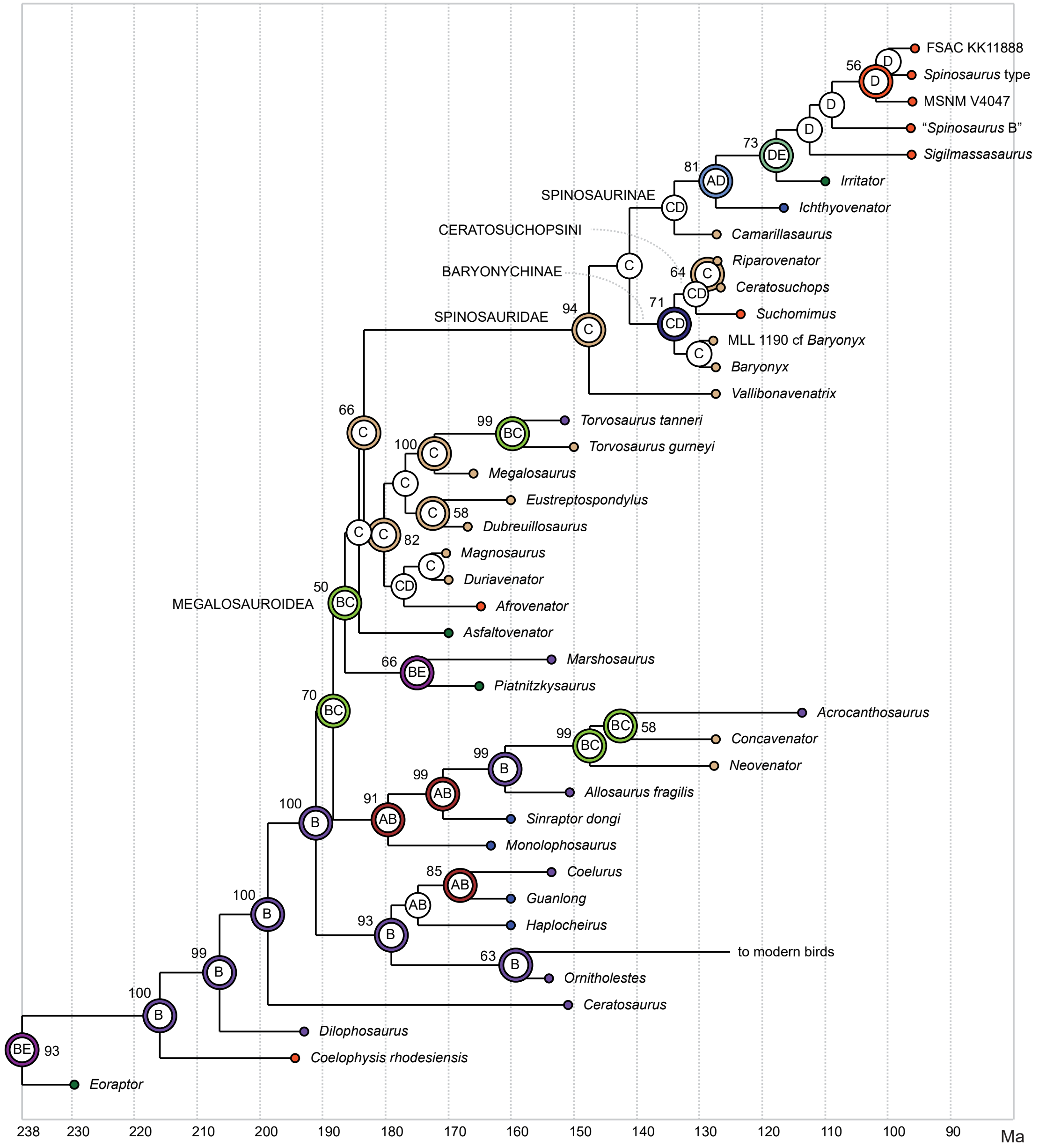

S1. Time-calibrated phylogeny of theropod relationships based on the Maximum Clade Credibility Tree inferred by the Bayesian analysis. Numbers at nodes represent posterior probability values >50%. Letters at nodes refer to the most likely ancestral area reconstructed. Colored circles indicate ancestral area inference with frequency >50%. Abbreviations: A, Asia; B, North America; C, Europe; D, Africa; E, South America.

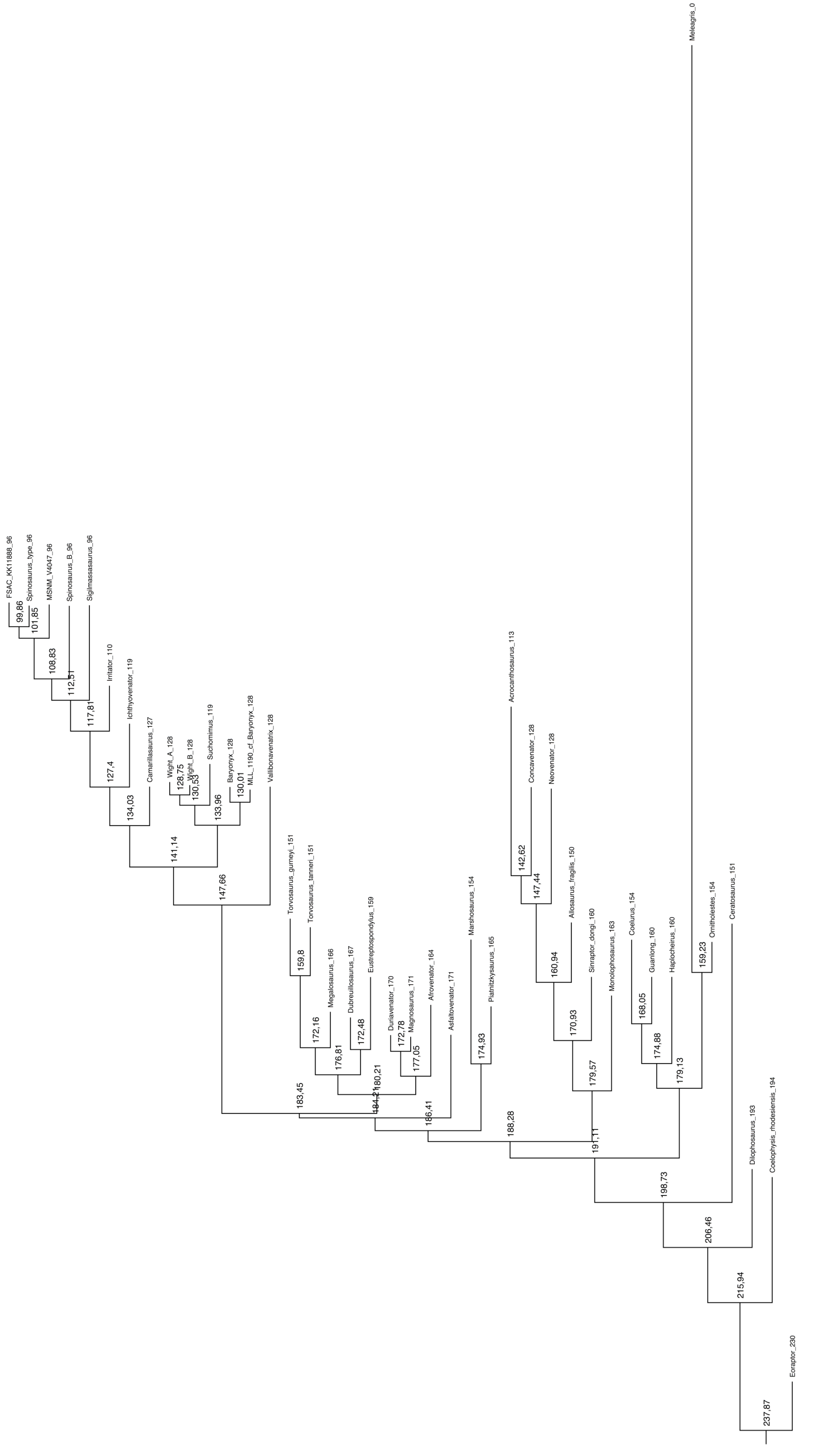

S2. Time-calibrated phylogeny of theropod relationships based on the Maximum Clade Credibility Tree inferred by the Bayesian analysis, showing age of node divergences.

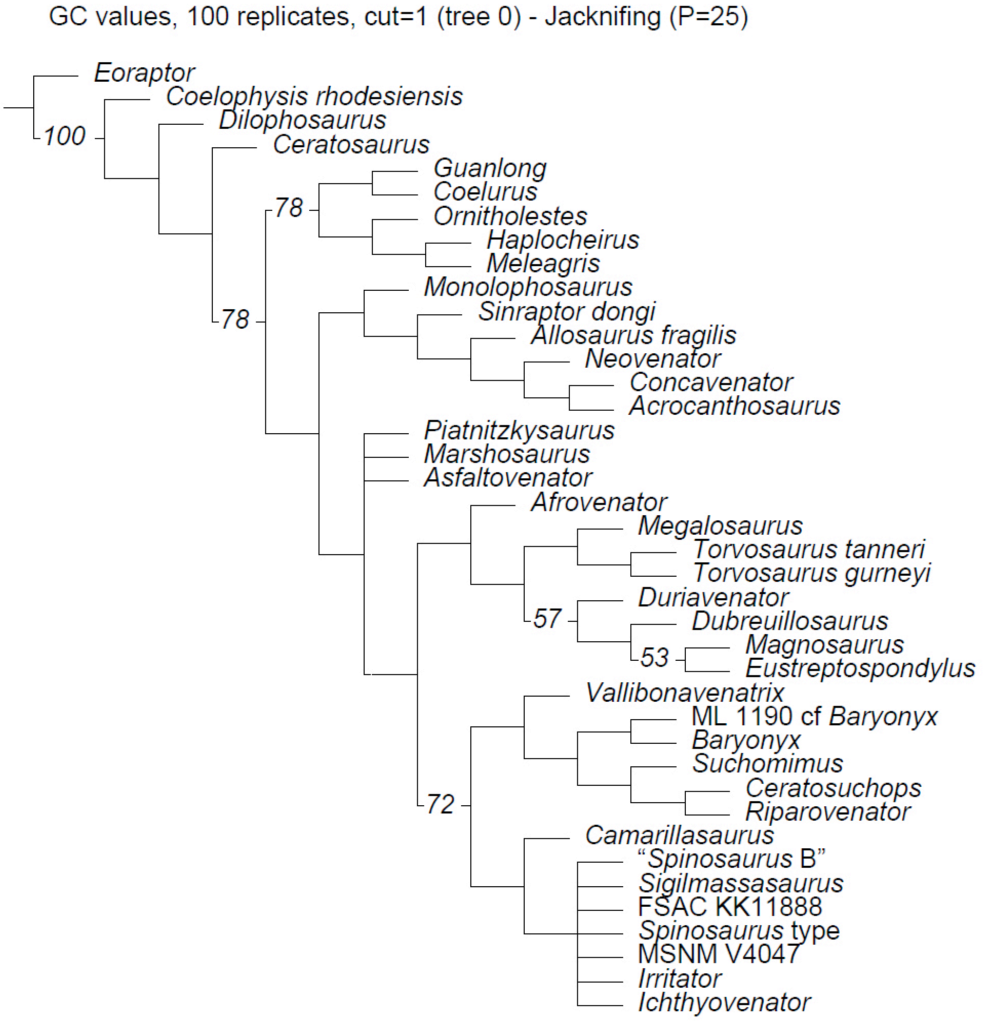

S3. Jackknife resampling for nodal support. Numbers at nodes indicate jackknife values in excess of 50%.

5 References

1 Charig, A. J. & Milner, A. C. *Baryonyx*, a remarkable new theropod dinosaur. *Nature* **324**, 359-361 (1986).

2 Charig, A. J. & Milner, A. C. *Baryonyx walkeri*, a fish-eating dinosaur from the Wealden of Surrey. *Bulletin-Natural History Museum Geology Series* **53**, 11-70 (1997).

3 Evers, S. W., Rauhut, O. W., Milner, A. C., McFeeters, B. & Allain, R. A reappraisal of the morphology and systematic position of the theropod dinosaur *Sigilmassasaurus* from the "middle" Cretaceous of Morocco. *PeerJ* **3**, e1323 (2015).

4 Hendrickx, C., Mateus, O. & Buffetaut, E. Morphofunctional Analysis of the Quadrate of Spinosauridae (Dinosauria: Theropoda) and the Presence of Spinosaurus and a Second Spinosaurine Taxon in the Cenomanian of North Africa. *PLoS One* **11** (2016).

5 Hendrickx, C., Mateus, O., Araújo, R. & Choiniere, J. The distribution of dental features in non-avian theropod dinosaurs: Taxonomic potential, degree of homoplasy, and major evolutionary trends. *Palaeontologia Electronica* **22** (2019).

6 Mateus, O., Araujo, R., Natário, C. & Castanhinha, R. A new specimen of the theropod dinosaur *Baryonyx* from the early Cretaceous of Portugal and taxonomic validity of Suchosaurus. *Zootaxa* **2827** (2011).

7 Sánchez-Hernández, B. & Benton, M. J. Filling the ceratosaur gap: A new ceratosaurian theropod from the Early Cretaceous of Spain. *Acta Palaeontologica Polonica* **59**, 581-600 (2014).

8 Rauhut, O. W., Canudo, J. I. & Castanera, D. in *Program and Abstracts XVII Conference of the EAVP* (ed European Association of Vertebrate Paleontologists (EAVP)) 96 (2019).

9 Samathi, A., Sander, P. M. & Chanthasit, P. A spinosaurid from Thailand (Sao Khua Formation, Early Cretaceous) and a reassessment of Camarillasaurus cirugedae from the Early Cretaceous of Spain. *Historical Biology*, 1-15 (2021).

10 Allain, R., Xaisanavong, T., Richir, P. & Khentavong, B. The first definitive Asian spinosaurid (Dinosauria: Theropoda) from the early cretaceous of Laos. *Naturwissenschaften* **99**, 369-377 (2012).

11 Sues, H. D., Frey, E., Martill, D. M. & Scott, D. M. *Irritator challengeri*, a spinosaurid (Dinosauria : Theropoda) from the Lower Cretaceous of Brazil. *Journal of Vertebrate Paleontology* **22**, 535-547, doi:Doi 10.1671/0272-4634(2002)022[0535:Icasdt]2.0.Co;2 (2002).

12 Sales, M. A. F. & Schultz, C. L. Spinosaur taxonomy and evolution of craniodental features: Evidence from Brazil. *PLOS ONE* **12**, e0187070, doi:10.1371/journal.pone.0187070 (2017).

13 Russell, D. A. Isolated dinosaur bones from the Middle Cretaceous of the Tafilalt, Morocco. *Bulletin du Muséum national d'Histoire naturelle, 4ème série–section C–Sciences de la Terre, Paléontologie, Géologie, Minéralogie* **18** (1996).

14 Dal Sasso, C., Maganuco, S., Buffetaut, E. & Mendez, M. A. New information on the skull of the enigmatic theropod *Spinosaurus*, with remarks on its size and affinities. *Journal of Vertebrate Paleontology* **25**, 888-896, doi:10.1671/0272-4634(2005)025[0888:NIOTSO]2.0.CO;2 (2005).

15 Stromer, E. Ergebnisse der Forschungsreisen Prof. E. Stromers in den Wüsten Ägyptens. II. Wirbeltierreste der Baharîje Stufe (unterstes Cenoman). 3. Das Original des Theropoden *Spinosaurus aegyptiacus* nov. gen., nov. spec. . *Abhandlungen der Königlich Bayerischen Akademie der Wissenschaften, Mathematisch-physikalische Klasse Abhandlung* **28** (1915).

16 Smith, J. B., Lamanna, M. C., Mayr, H. & Lacovara, K. J. New information regarding the holotype of *Spinosaurus aegyptiacus*. *Journal of Paleontology* **80**, 400-406 (2006).

17 Ibrahim, N. *et al.* Tail-propelled aquatic locomotion in a theropod dinosaur. *Nature* **581**, 67-70 (2020).

18 Ibrahim, N. *et al.* Semiaquatic adaptations in a giant predatory dinosaur. *Science* **345**, 1613-1616 (2014).

19 Stromer, E. Ergebnisse der Forschungsreisen Prof. E. Stromers in den Wüsten Ägyptens. II. Wirbeltier-Reste der Baharije-Stufe (unterstes Cenoman). 13. Dinosauria. *Abhandlungen der Bayer- ischen Akademie der Wissenschaften Mathematisch-naturwissen- schaftliche Abteilung* **22**, 1–79 (1934).

20 Sereno, P. C. *et al.* A long-snouted predatory dinosaur from Africa and the evolution of spinosaurids. *Science* **282**, 1298-1302 (1998).

21 Lipkin, C., Sereno, P. C. & Horner, J. R. The furcula in *Suchomimus tenerensis* and *Tyrannosaurus rex* (Dinosauria: Theropoda: Tetanurae). *Journal of Paleontology* **81**, 1523-1527 (2007).

22 Smith, N. D. *et al.* A *Megaraptor*-like theropod (Dinosauria: Tetanurae) in Australia: support for faunal exchange across eastern and western Gondwana in the Mid-Cretaceous. *Proceedings of the Royal Society B: Biological Sciences* **275**, 2085-2093 (2008).
23 Ibrahim, N. *et al.* Geology and paleontology of the Upper Cretaceous Kem Kem Group of eastern Morocco. *ZooKeys* **928**, 1 (2020).
24 Malafaia, E. *et al.* A new spinosaurid theropod (Dinosauria: Megalosauroidea) from the upper Barremian of Vallibona, Spain: Implications for spinosaurid diversity in the Early Cretaceous of the Iberian Peninsula. *Cretaceous Res* **106**, 104221 (2020).
25 Chiarenza, A. A. & Cau, A. A large abelisaurid (Dinosauria, Theropoda) from Morocco and comments on the Cenomanian theropods from North Africa. *PeerJ* **4**, e1754 (2016).
26 McFeeters, B. New mid-cervical vertebral morphotype of Spinosauridae from the Kem Kem Group of Morocco. *Vertebrate Anatomy Morphology Palaeontology* **8** (2020).
27 Bertin, T. A catalogue of material and review of the Spinosauridae. *PalArch's Journal of Vertebrate Palaeontology* **7**, 1-39 (2010).
28 Kellner, A. W., Azevedo, S. A., Machado, E. B., Carvalho, L. B. d. & Henriques, D. D. A new dinosaur (Theropoda, Spinosauridae) from the Cretaceous (Cenomanian) Alcântara Formation, Cajual Island, Brazil. *Anais da Academia Brasileira de Ciências* **83**, 99-108 (2011).
29 Carrano, M. T., Benson, R. B. & Sampson, S. D. The phylogeny of Tetanurae (Dinosauria: Theropoda). *Journal of Systematic Palaeontology* **10**, 211–300 (2012).
30 Buffetaut, E. & Ouaja, M. A new specimen of *Spinosaurus* (Dinosauria, Theropoda) from the Lower Cretaceous of Tunisia, with remarks on the evolutionary history of the Spinosauridae. *Bulletin de la Société géologique de France* **173**, 415-421 (2002).
31 Benyoucef, M. *et al.* Overabundance of piscivorous dinosaurs (Theropoda: Spinosauridae) in the mid-Cretaceous of North Africa: The Algerian dilemma. *Cretaceous Res* **55**, 44-55 (2015).
32 Cau, A. The assembly of the avian body plan: a 160-million-year long process. *Bollettino della Società Paleontologica Italiana* **57**, 1-25 (2018).
33 Dal Sasso, C., Maganuco, S. & Iurino, D. A. in *Second North African Vertebrate Palaeontology Congress - NAVEP2.* 26.
34 Bakker, R. T., Williams, M. & Currie, P. J. *Nanotyrannus*, a new genus of pygmy tyrannosaur, from the latest Cretaceous of Montana. *Hunteria* **1**, 1 (1988).
35 Russell, D. A. Ostrich dinosaurs from the Late Cretaceous of western Canada. *Canadian Journal of Earth Sciences* **9**, 375-402 (1972).
