# Supplementary material for: New spinosaurids from the Wessex Formation (Early Cretaceous, UK) and the European origins of Spinosauridae
Source: Sci Rep. 2021 Sep 29;11:19340. doi: 10.1038/s41598-021-97870-8 (PMC8481559; doi:10.1038/s41598-021-97870-8)
Supplement: Supplementary file 2 — Supplementary Information 2. [file 41598_2021_97870_MOESM2_ESM.pdf]

Group	#	Character statements (character polarity arbitrary in this context; parentheses containing "Ch. XX" refer to equivalent characters used in our phylogenetic analyses)	*Ceratosuchops*	*Riparovenator*	*Baryonyx* type	*cf. Suchomimus***	Caveats and points of consideration
**Unique characters observed in the Wessex Fm. specimens**							
Ceratosuchops	1	Basioccipital, width and position of the subcondylar recess: mediolaterally narrow and ventrally restricted (surface directly below condyle convex) (0); mediolaterally wide (~2/3 condyle width) and dorsoventrally tall (recess reaches the occipital condyle neck) (1) (see also Ch. 1191).	0	1	1	1	Reversal of the wide megalosaur condition. Ambiguous due to individual/ontogenetic variability of pneumatic features (Chure and Madsen, 1996; Witmer 1990, 1997a, b; Sampson and Witmer 2007; Witmer and Ridgley 2010; Carr, 2020). Surface below condyle develops from convex ("large stage 1" specimens) to concave ("stage 3 specimens") during *Gorgosaurus* growth series (Carr, 1999: 519).
	2	Basisphenoid, excavation of the oval scars: shallow depressions (0); elongate, narrow sulci (1).	1	0	n/a	0	Oval scar excavation apomorphic for Ceratosuchopsini (see main text; row 37), *Ceratosuchops* could simply represent individual variation of this trait (albeit arguably extreme in terms of morphological difference).
	3	Basisphenoid, width of the interbasipterygoidal web: thin (0); thick (1).	1	0	0	0	Ambiguous if individual variability affecting pneumatic features bordering it; Web becomes thicker during *Tyrannosaurus* ontogeny (Carr, 2020: ch 1050 – blade-like in young adults, long and convex in adults), although Wealden Supergroup spinosaurids do not display blade-like morphology.
	4	Supraoccipital, dorsal process, posterior surface in coronal view (below more dorsally positioned sulcus): gently curved (0); V-shaped or produces a midline ridge (1).	0	1	1	1	Variation in ridge presence and development is present in various coelurosaurs and may characterise early members of the clade (Bever et al., 2013). Midline ridge present in *Irritator* also (Sues et al., 2002).
Riparovenator	5	Premaxilla, shape of pm1tooth/alveolus relative to pm2: pm1 at least half the size of pm2 (0); pm1 less than half the size of pm2 (1).	0	1	0	0	Heterodonty exhibited by spinosaurids generally; possibly too variable to be taxonomically useful (C. Hendrickx, pers. comm. 2021).
	6	Frontal, prefrontal contact in dorsal view: prefrontal articulates along anterior surface of the frontal postorbital process, no notch (0); prefrontal does not contact frontal postorbital process, notch present (1) (see also Ch. 1498)	0	1	0	0	Possibly an immature trait displaying incomplete closure in *Riparovenator*; Orbital notch closure ontogenetically and individually variable in *Tyrannosaurus* (Carr, 2020: Ch 90)
	7	Prootic, visibility of the facial nerve (CN VII) foramen in lateral view: foramen visible (0); foramen largely obscured by thick otosphenoidal crest (1).	0	1	0	0	
	8	Basioccipital, depth of the subcondylar recess: shallow, depth less than 1/5 of recess mediolateral width (0); deep, depth over 1/3 recess mediolateral width (1).	0	1	0	0	Potentially ambiguous due to variability of pneumatic features (see above); surface ventral to the condyle convex to concave (in *Daspletosaurus)* (Carr, 1999: 519). However, excavation of "ventral plate" (deep vs shallow) helped distinguish *Daspletosaurus horneri* and *D. torosus* (Carr et al., 2017); whilst "subcondylar recess" depth changes during *Tyrannosaurus* ontogeny (Carr, 2020: Ch 111 – deep in large juveniles, shallow by young adult stage), these refer to different set of pneumatic features not situated directly ventral to condyle (see also Witmer and Ridgley, 2009).
**Potentially obsolescent/ambiguous "unique" characters in *Ceratosuchops* or *Riparovenator***							
Ceratosuchops	9	Premaxillae, antenarial tuberosities (located on the anterior margin of external narial margin): absent (0); present (1).	1	?	0	0	
	10	Premaxilla, subnarial process, length: subequal to or more than (0); less than (1) the length of the buccal margin of the premaxilla (Ch. 752)	1	?	?	0	Process lengths generally variable (Molnar 1990), longer process in *Suchomimus* affects slightly larger specimen; Ontogenetic/individual variation observed in *Tyrannosaurus* (Carr, 2020: Ch 265 – some adults display immature "short" state, despite mature state already acquired by some young adult morphs).
	11	Skull roof, path of the frontoparietal suture: V-shaped (0); "step"-like (intitally transverse medially, before trending posteriorly a short distance and regaining a transverse orientation laterally) (1); sigmoidal (2)	0	1	?	2	Frontoparietal suture changes observed in tyrannosaurid ontogeny (Carr, 2020: Ch92 – some adult *Tyrannosaurus* display immature"wedge-shaped" state, vice versa for some large juveniles; Carr and Williamson, 2004); however frontoparietal contact differences helped distinguish *Daspletosaurus horneri* and *D. torosus* (Carr et al., 2017).
	12	Otoccipitals, angle of projection of paroccipital proccesses: laterally (sub-horizontally) (0); posterolaterally (1).	1	?	0	?	Variation in projection angle apparently observed in some tyrannosaurid taxa (development from more posterior to more lateral projection) (Bever et al., 2013: 19).
	13	Otoccipitals, anterior margin of the facet on the dorsal paroccipital process: angular, margin projects anteriorly (0); curved, minimal anterior projection (1).	1	0*	0*	?	*Potentially ambiguous as the right otoccipitals in *Baryonyx* and *Riparovenator* are missing lateral part of the process; unable to test for intra-individual variation in facet shape.
	14	Basisphenoid, basisphenoid recess, dorsoventral extent: recess short and does not extend far under basioccipital (0); extends dorsally under basioccipital apron (1)	0	1	1	?	Ambiguous due to individual variability of pneumatic features. See Duffeau (2011) for *Baryonyx*.
	15	Cultriform process, direction of projection of process tip (relative to the horizontally oriented skull roof): anteriorly (0); anteroventrally (1).	0	1	?	?	Strongly arched processes observed in *Tyrannosaurus* ontogeny (Witmer and Ridgley, 2009 – on basis CMNH 7541 "*Nanotyannus*" is a immature morph of *Tyrannosaurus*) and *D. torosus* (Carr, 1999: 507).
	16	Occiput, crista tuberalis (=metotic strut), mediolateral width across opposing cristae in posterior view: less than (0); more than (1) 1/2 the dorsoventral depth of the braincase from the dorsal tip of the supraoccipital to the ventral tip of the basal tubera (ch 1014).	0	?	1	?	Possibly influenced by ontogenetic variation in paraoccipital process projection (see also #12)
Riparovenator	17	Nasal, cruciform process, shape of posterodorsal margin (i.e. dorsal margin of the frontal process) in lateral view: straight (0); curved (1).	?	1	0	?	
	18	Skull roof, path of the frontoparietal suture: V-shaped (0); "step"-like (intitally transverse medially, before trending posteriorly a short distance and regaining a transverse orientation laterally) (1); sigmoidal (2)	0	1	?	2	Replicate (see #11)
	19	Dorsum sellae, shape of the dorsal margin in anterior view: V-shaped (0); sub horizontal (1).	0*	1	0	?	*based on mirroring the undamaged right side of the dorsum sellae.
	20	Cultriform process, direction of projection of process tip (relative to the horizontally oriented skull roof): anteriorly (0); anteroventrally (1).	0	1	?	?	Replicate (see #15)
	21	Cultriform process, subdivsion of the subsellar recess by thin lamina: absent (0); present (1)	?	1	?	?	Pneumatic features generally variable, comparative sample unknown for baryonychines.
	22	Basisphenoid, basipterygoid process, shape of lateral margin in ventral view: convex (0); concave (1).	0	1	?	0	
	23	Basiphenoid, basipterygoid process, exposure of the ventral surface in lateral view: thick (0); reduced (1).	0	1*	?	0	*minor damage to left process' ventral margin
	24	Caudal vertebrae, anteroposterior width of anterior neural spines: narrow (0); wide (1).	?	0	1*	1	*Fragmentary evidence suggest some breadth in *Baryonyx* (Charig and Milner, 1990, 1997); Comparisons potentially affected by non-overlapping elements. Anterior caudal neural spines also anteroposteriorly narrow in *Ichthyovenator*.
	25	Caudal vertebrae, anterior neural arches, ventral rib laminae: absent (0); present (1) (Ch. 358)	?	0	1	?	Not unique as *Vallibonavenatrix* is also scored the same (taxon recovered as baryonychine in parsimony analysis.); Comparisons potentially affected by non-overlapping elements e.g. more distal anterior *Ichthyovenator* caudals appear to lack ventral rib lamina relative to laminated anteriormost element.
	26	Caudal vertebrae, anterior neural arches, hyposphene: absent (0); present (1) (Ch. 359).	?	0	1	?	Not unique as *Vallibonavenatrix* is also scored the same (taxon recovered as baryonychine in parsimony analysis.);
	27	Caudal vertebrae, constriction of the centrum midpoint: less than (0) or more than (1) 40% anterior facet width.	?	1	0	?	Possibly allometric, state 1 affects larger specimen; overlap of vertebrae uncertain.
**Further Comparisons: Characters shared between both Wessex Fm. specimens but not other baryonychines**							
Ceratosuchops + *Riparovenator*	28	Premaxilla, paradental plates: absent (0); present (1)	0*	0*	1	1	*Possibly taphonomic.
	29	Premaxilla, interalveolar space between 3rd and 4th alveoli, mesiodistal diameter: less (0); subequal or more (1) than ½ of 4th alveolus mesiodistal diameter (Ch. 512)	0	0	1	1	Interalveolar space can be variable in baryonychines, and heterodonty exhibited by spinosaurids generally. State 1 present in juvenile spinosaurid premaxillae (Lakin and Longrich, 2019), suggesting character not influenced by ontogeny (but sample size low).
	30	Basioccipital, ventral occipital condyle margin in ventral view: V-shaped (0); gently curved (1)	1	1	0	0	Ventral condyle shape ontogenetically and individually variable in tyrannosaurids (Carr, 2020: Ch 1020 – immature state observed in some adult *Tyrannosaurus*). Left ventrolateral margin in cf. *Suchomimus* possibly slighty damaged.
**Further Comparisons: Characters shared between both Wessex Fm. specimens and *Suchomimus* but not *Baryonyx***							
	31	Enamel ornamentation*: lingually only (0); labially and lingually (1).	1	1	0	1	*Enamel ornamentation variable and potentially of limited significance (Fowler, 2007; Hendrickx et al., 2016, 2019).

Ceratosuchops + Riparovenator + cf. Suchomimus	32	Frontal, postorbital facet, anterior depth: less (0); more (1) than 2/5 facet length (Ch. 1053).	1	1	0	1	Cranial facets may be generally variable (Molnar, 1990), deeper facets affect larger specimens; Ontogeny/individual variation affects postorbital frontal process shape (and thus frontal postorbital facet shape, which accomodates the process) in *Tyrannosaurus* (Carr, 2020: Ch 68, 69 – some young adults and adults display the immature "shorter" state re: facet height and length).
	33	Frontal, postorbital facet, deep longitudinal slot for postorbital: absent (0); present (1) (Ch. 1761).	1	1	0	1	Slot within frontal postorbital facet develops ontogenetically in *Tyrannosaurus* (Carr, 2020: Ch 919 – groove acquired by young adult stage), smallest specimen (*Baryonyx*) does not produce slot.
	34	Frontal, anterior margins of supratemporal fossa: poorly developped with little curvature (0) sharp and laterally curving (1).	1	1	0	1	Margins change shape (Carr, 1999) and become increasingly prominent in tyrannosaurids growth series but may be individually variable also (Carr, 2020: Ch 935 – some young adults and adults stages display immature state); Trait affects larger baryonychine specimens.
	35	Basioccipital, ventral occipital condyle margin in posterior view: V-shaped, margins converge towards midline (0); gently curved (1).	1	1	0	1	State 0 reminiscent of immature tyrannosaurid state (Carr, 1999; Carr, 2020: Ch 1018 – mature "reniform" state acquired by young adult stage in *Tyrannosaurus*).
	36	Basioccipital, contribution to foramen magnum margin: large, exoccipitals widely separated (0); reduced, exoccipitals closely placed (1) (Ch. 974).	1	1	0	1	Closely appressed exoccipitals also in *Irritator* (S. Evers, pers. comms. 2021). Some variation in at least one tyrannosaurid (reduced contact in an immature *Gorgosaurus*, space normally wider in tyranosaurids – Bever et al 2013: 25). More tangentially, basioccipital contribution to foramen magnum increases in *Psittacosaurus* ontogeny (hatchling and juveniles: 15%, adults 30%) (Bullar et al., 2019) but may decrease in some ceratopsids (Longrich and Field, 2012: Ch 4).
	37	Basisphenoid, excavation of the collateral scars placed lateral to middle depression: absent (0); present and distinct on both sides (1) (Ch. 510).	1	1	0	1	Does affect a probably muscular insertion point (thus potential for individual variation in attachment morphology); "dished"/"concave" scars seen in adult tyrannosaurids (Carr, 1999).
	38	Basisphenoid, median recess, depth: shallow (0); deep (1) (Ch. 1566).	1	1	0	1	Pneumatic features generally variable (see above).
	39	Basisphenoid, ridges (cf. cristae ventrolateralis of some) binding the median recess laterally: absent (0); present (1).	1	1	0	1	Probably redundant with scar excavation (see row 41); Increase in ridge prominence throughout *Tyrannosaurus* ontogeny (Carr, 2020: Ch 1044), however immature state in latter already contains ridged margins.
	40	Basisphenoid, posteroventral edge of interbasipterygoidal web in sagittal section: sharp (0); rounded (1).	1	1	0	1	Web becomes thicker and convex during *Tyrannosaurus* ontogeny (blade-like in younger morphs; Carr, 2020: ch 1050).
	41	Otoccipial, number of cranial nerve foramina lateral to occipital condyle: two (0); three (1).	1	1	0	1	Number can be variable in *Tyrannosaurus* (Bever et al., 2013: 22), unknown if number of foramina phylognetically informative at this stage (Brusatte et al., 2010).
	42	Prefrontal, presence of a boss-like process: absent (0); present (1)	1	1	?*	1	*Prefrontal recovered but potentially damaged.
**Further Comparisons: Characters shared by at least one Wessex Fm. specimen and *Suchomimus*, but not *Baryonyx***							
Ceratosuchops + cf. Suchomimus	43	Premaxilla, narial fossa: absent (0); present (1) (Ch. 709).	1	?	0	1	Limited data on narial fossa variation in theropods. Tangentially, variation observed in *Gavialis* (Hone et al., 2020).
	44	Basioccipital, subcondylar recess bordered laterally by mediolaterally thick crests: absent (0); present (1).	0	1	1	0	Individual and ontogenetic varition observed in *Tyrannosaurus* "ascending scars" (Carr, 2020: Ch 1024 –some adult morphs display immature "prominent" state).
	45	Parietal, posterior rise of the nuchal crest in lateral view (angle measured in lateral view with the skull roof held horizontally; the vertex of the angle is located on the capitate process of the laterosphenoid (or preserved equivalent); the first ray projects to the dorsal nuchal crest; the second ray projects posteriorly): ≥ (0), or < (1) 45º.	1	?*	0	1	*Parietal damaged however general trend is likely comparable to that of cf. *Suchomimus* (42º) and *Ceratosuchops* (44º) rather than *Baryonyx* (70º).
**Further Comparisons: Characters shared between both Wessex Fm. specimen and *Baryonyx* but not *Suchomimus***							
Ceratosuchops + Riparovenator + Baryonyx	46	Orbital margin, exposure of the orbital fossae in lateral view: orbital fossae visible (0); dorsal portion of orbital fossae partially obscured by overhang of the orbital rim (1).	0	0	0	1*	*Taphonomic in c.f. *Suchomimus*?
	47	Basioccipital, proportions relative to basisphenoid (measured along midline ventral to occipital condyle to interbasipterygoidal web), in posterior view: basioccipital contribution shorter (0) or longer (1) than basisphenoid contribution.	0	0	0	1	
	48	Occiput, relationship between of basisphenoid oval scars and ventral portion of basioccipital apron: apron situated between oval scars (0); ventral plate overlaps oval scars (1)	1	1	1	0	
	49	Basioccipital, foramina situated ventrolaterally occipital condyle: absent (0); present (1).	0	0	0	1*	*Ambiguous as right side of *Suchomimus* not well exposed in available imagery. Observable foramina do not relate to foramina for X-XII.
**Further Comparisons: Characters shared by at least one Wessex Fm. specimen and *Baryonyx* but not *Suchomimus***							
Ceratosuchops + Baryonyx	50	Premaxilla, subnarial process, slenderness: shorter than (0); longer than (1) 4 times its proximal depth (Ch. 697)	0	?	0	1	See also #10
	51	Premaxilla, dorsal midline: uncrested (0); crested (1)	0	?	0	1	
	52	Supraoccipital, dorsal process, width (measured between the foramina for the middle cerebral vein) relative to height (measured from a middle cerebral vein foramen to the tip of the dorsal process): width more than (0) or less than (1) 2/3 process height.	0	?	0	1	State 1 affects largest specimen, character could be allometrically variable.
	53	Supraoccipital, dorsal process, posterodorsal margins: in line with (0) or overhang (1) the rest of the dorsal process.	0	?	0	1*	*Overhang potentially related to the development of cervical musculature – process on dorsal end of supraoccipital identified as the tendinous insertion for the m. spinalis capitis in *Tyrannosaurus* (Tsuihiji 2010).
Riparovenator + Baryonyx	54	Basioccipital, subcondylar recess bordered laterally by mediolaterally thick crests: absent (0); present (1).	0	1	1	0	Replicate (see #44)

** cf. *Suchomimus* scores based on hypodygm of several specimens inc. MNN GDF 500, 501, 214

References

Bever, G.S., Brusatte, S.L., Carr, T.D., Xu, X., Balanoff, A.M. and Norell, M.A. 2013. The braincase anatomy of the Late Cretaceous dinosaur Alioramus (Theropoda: Tyrannosauroidea). Bulletin of the American Museum of Natural History, 2013(376), pp.1-72.

Brusatte, S.L., Chure, D.J., Benson, R.B. and Xu, X. 2010. The osteology of *Shaochilong maortuensis*, a carcharodontosaurid (Dinosauria: Theropoda) from the Late Cretaceous of Asia. Zootaxa, 2334(1), pp.1-46.

Carpenter, K. 2010. Variation in a population of Theropoda (Dinosauria): *Allosaurus* from the Cleveland-Lloyd Quarry (Upper Jurassic), Utah, USA. Paleontological research, 14(4), pp.250-259.

Carr, T.D. 1999. Craniofacial ontogeny in tyrannosauridae (Dinosauria, Coelurosauria). Journal of vertebrate Paleontology, 19(3), pp.497-520.

Carr, T.D. 2020. A high-resolution growth series of *Tyrannosaurus rex* obtained from multiple lines of evidence. PeerJ, 8, p.e9192.

Carr, T.D. and Williamson, T.E. 2004. Diversity of late Maastrichtian Tyrannosauridae (Dinosauria: Theropoda) from western North America. Zoological Journal of the Linnean Society, 142(4), pp.479-523.

Carr, T. D., Varricchio,D. J., Sedlmayr, J. C., Roberts, E. M., and Moore, J. R. 2017. A new tyrannosaur with evidence for anagenesis and crocodile-like facial sensory system. Scientific Reports 7, pp. 1-11.

Charig, A.J. and Milner, A.C. 1990. The systematic position of *Baryonyx walkeri*, in the light of Gauthier's reclassification of the Theropoda. In: Dinosaur systematics, Approaches and Perspectives, K. Carpenter and P. J. Currie (eds), pp. 127-140.

Charig, A.J. and Milner, A.C. 1997. *Baryonyx walkeri*, a fish-eating dinosaur from the Wealden of Surrey. Bulletin-Natural History Museum Geology Series, 53, pp.11-70.

Chure, D.J. and Madsen Jr, J.H. 1996. Variation in aspects of the tympanic pneumatic system in a population of Allosaurus fragilis from the Morrison Formation (Upper Jurassic). Journal of Vertebrate Paleontology, 16(1), pp.63-66.

Duffeau, D. L. 2011. The Evolution of Cranial Pneumaticity in Archosauria: Patterns of Paratympanic Sinus Development. PhD Thesis, Ohio University.

Fowler, D. 2007. Recently rediscovered baryonychine teeth (Dinosauria: Theropoda): New morphologic data, range extension & similarity to Ceratosaurus. Journal of Vertebrate Paleontology 27(3), pp. 76A

Hendrickx, C., Mateus, O., Araújo, R. and Choiniere, J. 2019. The distribution of dental features in non-avian theropod dinosaurs: Taxonomic potential, degree of homoplasy, and major evolutionary trends. Palaeontologia Electronica, 22(3).

Hone, D., Mallon, J.C., Hennessey, P. and Witmer, L.M. 2020. Ontogeny of a sexually selected structure in an extant archosaur *Gavialis gangeticus* (Pseudosuchia: Crocodylia) with implications for sexual dimorphism in dinosaurs. PeerJ, 8, p.e9134.

Lakin, R.J. and Longrich, N.R. 2019. Juvenile spinosaurs (Theropoda: Spinosauridae) from the middle Cretaceous of Morocco and implications for spinosaur ecology. Cretaceous Research, 93, pp.129-142.

Molnar, R.E. 1990. Variation in theory and in theropods. In: Dinosaur systematics, Approaches and Perspectives, K. Carpenter and P. J. Currie (eds), pp. 71-79.

Sampson, S.D. and Witmer, L.M. 2007. Craniofacial anatomy of *Majungasaurus crenatissimus* (Theropoda: Abelisauridae) from the late Cretaceous of Madagascar. *Journal of Vertebrate Paleontology*, *27*(S2), pp.32-104.

Sues, H.D., Frey, E., Martill, D.M. and Scott, D.M. 2002. *Irritator challengeri*, a spinosaurid (Dinosauria: Theropoda) from the Lower Cretaceous of Brazil. Journal of Vertebrate Paleontology, 22(3), pp.535-547.

Tsuihiji, T. 2010. Reconstructions of the axial muscle insertions in the occipital region of dinosaurs: evaluations of past hypotheses on Marginocephalia and Tyrannosauridae using the Extant Phylogenetic Bracket approach. The Anatomical Record:

Advances in Integrative Anatomy and Evolutionary Biology, 293(8), pp.1360-1386.
Witmer, L.M., 1990. The craniofacial air sac system of Mesozoic birds (Aves). Zoological Journal of the Linnean Society, 100(4), pp.327-378.
Witmer, L. M. 1997a. Craniofacial air sinus systems, in: The Encyclopedia of Dinosaurs, P. J. Currie and K. Padian (eds.), Academic Press, New York, pp. 151– 159
Witmer, L.M. 1997b. The evolution of the antorbital cavity of archosaurs: a study in soft-tissue reconstruction in the fossil record with an analysis of the function of pneumaticity. Journal of Vertebrate Paleontology, 17(S1), pp.1-76.
Witmer, L.M. and Ridgely, R.C. 2009. New insights into the brain, braincase, and ear region of tyrannosaurs (Dinosauria, Theropoda), with implications for sensory organization and behavior. The Anatomical Record: Advances in Integrative Anatomy and Evolutionary Biology, 292(9), pp.1266-1296.
Witmer, L.M. and Ridgely, R.C. 2010. The Cleveland tyrannosaur skull (*Nanotyrannus* or *Tyrannosaurus*): new findings based on CT scanning, with special reference to the braincase. Kirtlandia, 57, pp.61-81.
