# Supplementary material for: New spinosaurids from the Wessex Formation (Early Cretaceous, UK) and the European origins of Spinosauridae
Source: Sci Rep. 2021 Sep 29;11:19340. doi: 10.1038/s41598-021-97870-8 (PMC8481559; doi:10.1038/s41598-021-97870-8)
Supplement: Supplementary file 3 — Supplementary Information 3. [file 41598_2021_97870_MOESM3_ESM.pdf]

Appendix 2: Additional imagery of the diagnostic and differentiating cranial features of the Wessex Fm. spinosaurids.

This appendix provides additional imagery of the diagnostic traits of *Ceratosuchops inferodios* and *Riparovenator milnerae*. Several distinguishing features are also imaged here, and comparisons are made to the condition in *Baryonyx* (where possible).

Contents

1	Diagnoses of the Wessex Fm. spinosaurids.	1
2	Imagery of diagnostic the traits of *Ceratosuchops inferodios*	2
3	Imagery of other distinguishing traits of *Ceratosuchops inferodios* relative to *Baryonyx walkeri* and/or *Riparovenator milnerae*	6
4	Imagery of the diagnostic traits of *Riparovenator milnerae*	10
5	Other distinguishing traits of *Riparovenator milnerae* relative to *Baryonyx walkeri* and/or *Ceratosuchops inferodios*	13

1. Diagnoses of the Wessex Fm. spinosaurid

Reiterated below are the diagnostic and distinguishing features of the Wessex Fm. spinosaurids. Traits in bold (and their associated numeration) are imaged in this appendix.

Ceratosuchops inferodios

Baryonychine distinguished by the presence of the following unique traits: **premaxillae displaying a pair of bilaterally located antenarial tuberosities (1)**; **narrow (reversal of the ancestral megalosauroid condition) and ventrally restricted subcondylar recess of the basioccipital (2)**; **oval scars of the basisphenoid excavated by deep, elongate sulci (3)**; **anteroposteriorly thick interbasipterygoidal web (4)**; **supraoccipital dorsal process possessing a gently curving posteroventral surface in coronal section (5)**.

This taxon can be further separated from other baryonychines by the following combination of traits: **presence of narial fossae on the premaxilla (6)** (as in cf. *Suchomimus* but not *Baryonyx*); short subnarial (maxillary) process of the premaxilla (as in *Baryonyx* but not cf. *Suchomimus*); lack of premaxillary sagittal crest (as in *Baryonyx* but not cf. *Suchomimus*); **curved anterior margin of the dorsal facet of the paroccipital process (7)** (angular in *Baryonyx* and probably *Riparovenator*); **posterolaterally directed paroccipital processes of the otoccipitals (8)** (more laterally directed in *Baryonyx*); **exoccipital components of the occipital condyle closely spaced (9)** (as in *Riparovenator* and cf. *Suchomimus* but not *Baryonyx*); **subcondylar recess lacking mediolaterally thick lateral crests (10)** (as in cf. *Suchomimus* but not *Baryonyx* or *Riparovenator*); relatively stout supraoccipital dorsal process (as in *Baryonyx* but not cf. *Suchomimus*); lack of a dorsal extension of the basisphenoid recess under the basioccipital apron (recess extends dorsally in *Baryonyx* and *Riparovenator*).

Riparovenator milnerae

Baryonychine distinguished by the presence of the following unique traits: **notched dorsal orbital margin between prefrontal and postorbital process of the frontal (1)**; **deeply inset facial nerve (CN VII) foramen that is largely obscured from lateral view (2)**; **deep subcondylar recess (3)** (depth over 1/3 of its mediolateral width; depth less than 1/5 in other baryonychines); **reduced exposure of ventral surface of the basipterygoid processes in lateral view (4)**.

This taxon can be further separated from other baryonychines by the following combination of traits: **curved dorsal margin of the frontal process of the nasal in lateral view (5)** (margin effectively straight in *Baryonyx*); **straight dorsal margin of the dorsum sellae (6)** (V-shaped in *Baryonyx* and *Ceratosuchops*); **exoccipital components of the occipital condyle closely spaced (7)** (as in *Ceratosuchops* and cf. *Suchomimus* but not *Baryonyx*); **mediolaterally thick crests bordering the subcondylar recess (8)** (as in *Baryonyx* but not *Ceratosuchops* or cf. *Suchomimus*); **lateral margins of the basipterygoid processes concave in ventral view (9)** (convex in *Ceratosuchops*).

2. Imagery of the diagnostic traits of *Ceratosuchops inferodios*

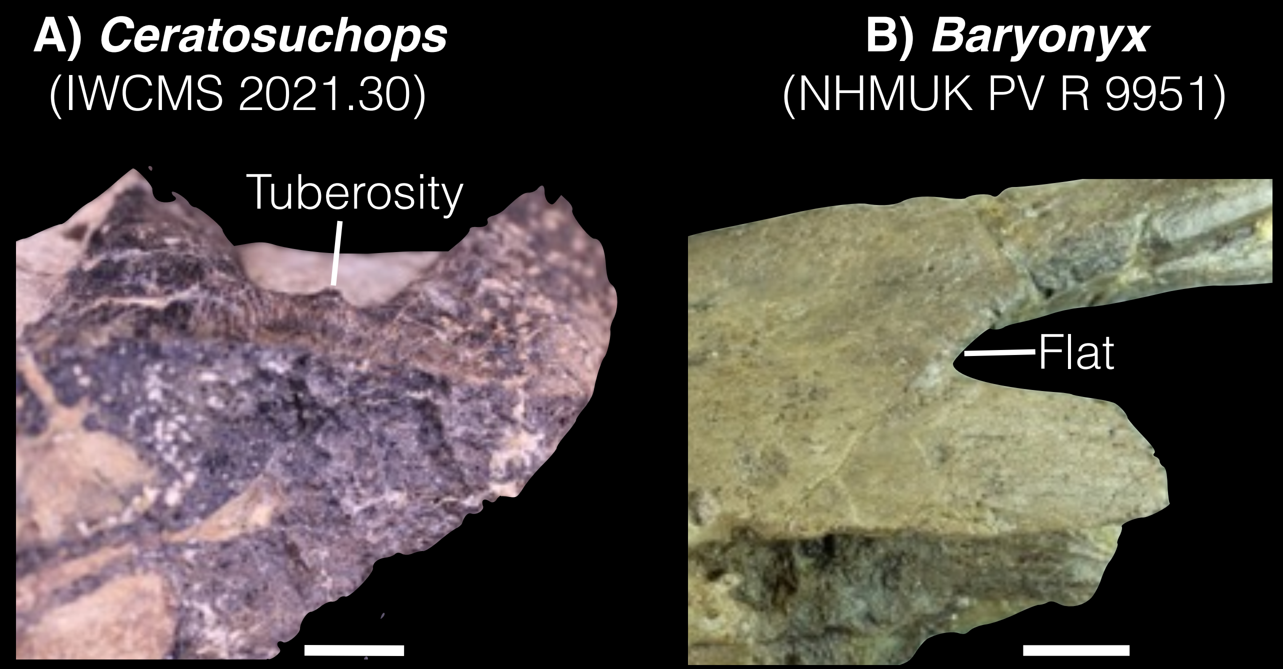

1. Presence of an antenarial tuberosity on the premaxilla. Premaxillae in A) right anterior-oblique view, B) left lateral view. Abbreviations: np, nasal process.Scale bars: A) 10mm, B) 20mm.

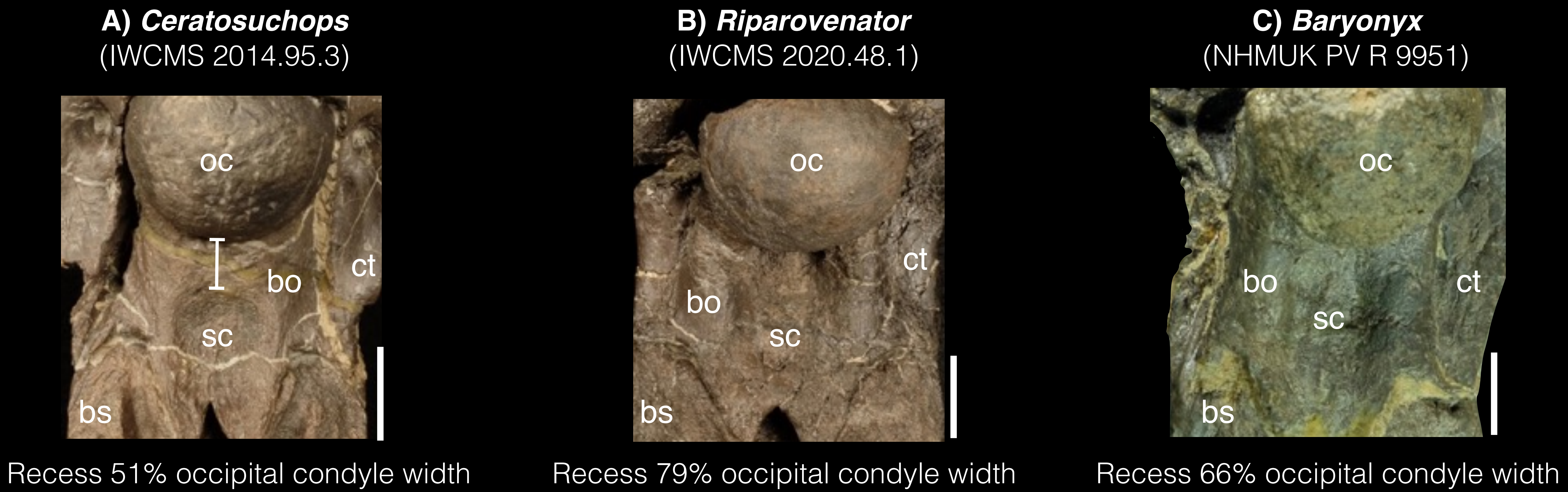

2. Presence of a narrow and ventrally restricted subcondylar recess. A–C) basicrania in posterior view. Note how the recess doesn't reach the occipital condyle neck in *C. infernodios*. Recess width measured between two highest points directly bordering the depression. Abbreviations: bo, basioccipital; bs, basisphenoid; ct: crista tuberalis; oc: occipital condyle; sc, subcondylar recess. Scale bars: 20mm

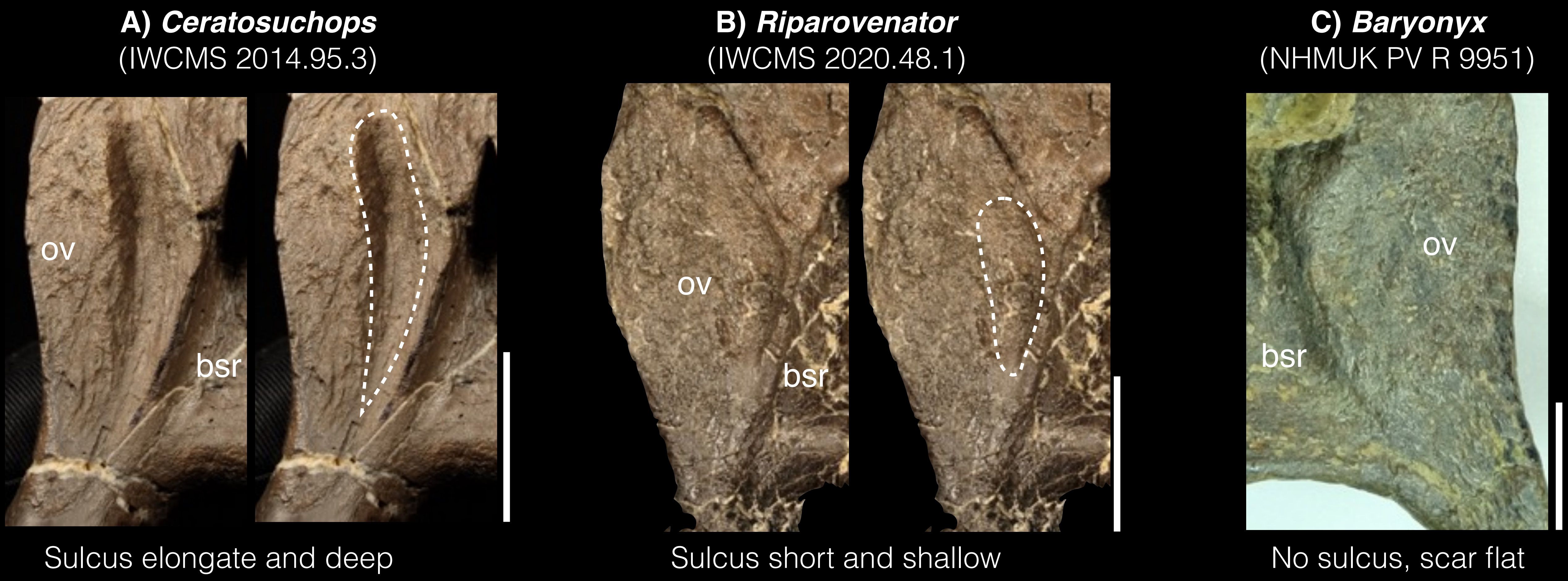

3. Presence of deep, elongate sulci excavating basisphenoid scars. Left (A, B) and right (C) basisphenoid oval scar in posterior view, A) and B) are stereopairs. Abbreviations: bsr, basisphenoid recess; ov, oval scar. Scale bars: 20mm.

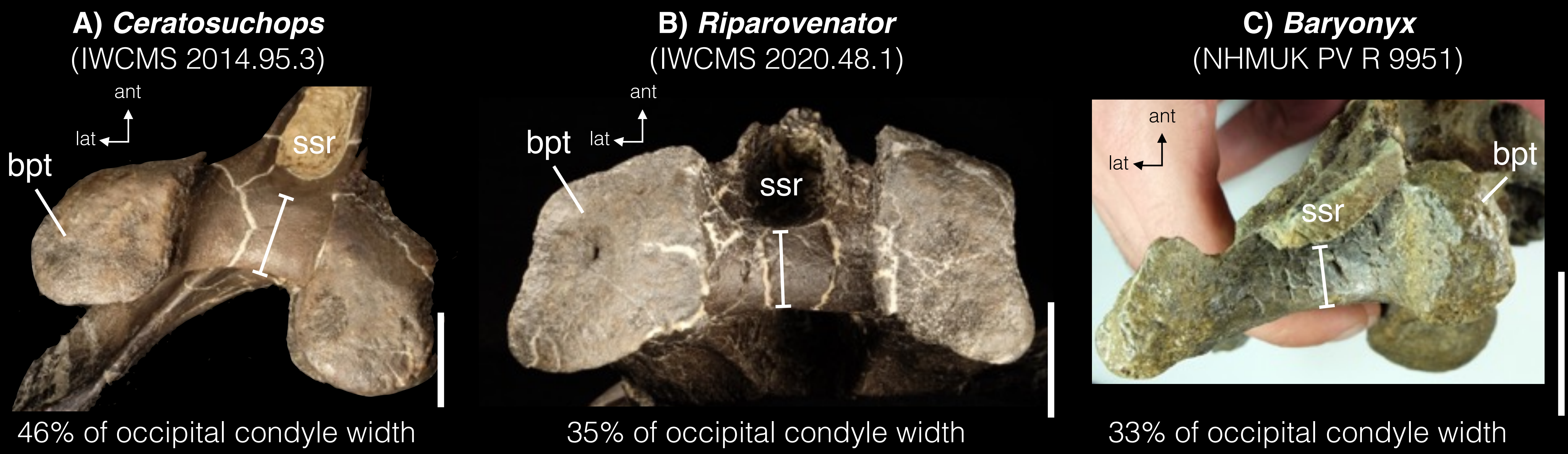

4. Presence of a relatively anteroposteriorly long interbasipterygoidal web. A–C) Basisphenoids in ventral view. Distance marked in A) 20.9mm, B) 16.3mm, C) 14.8 mm. Abbreviations: bpt, basipterygoid process; ssr, subsellar recess. Scale bars: 20mm.

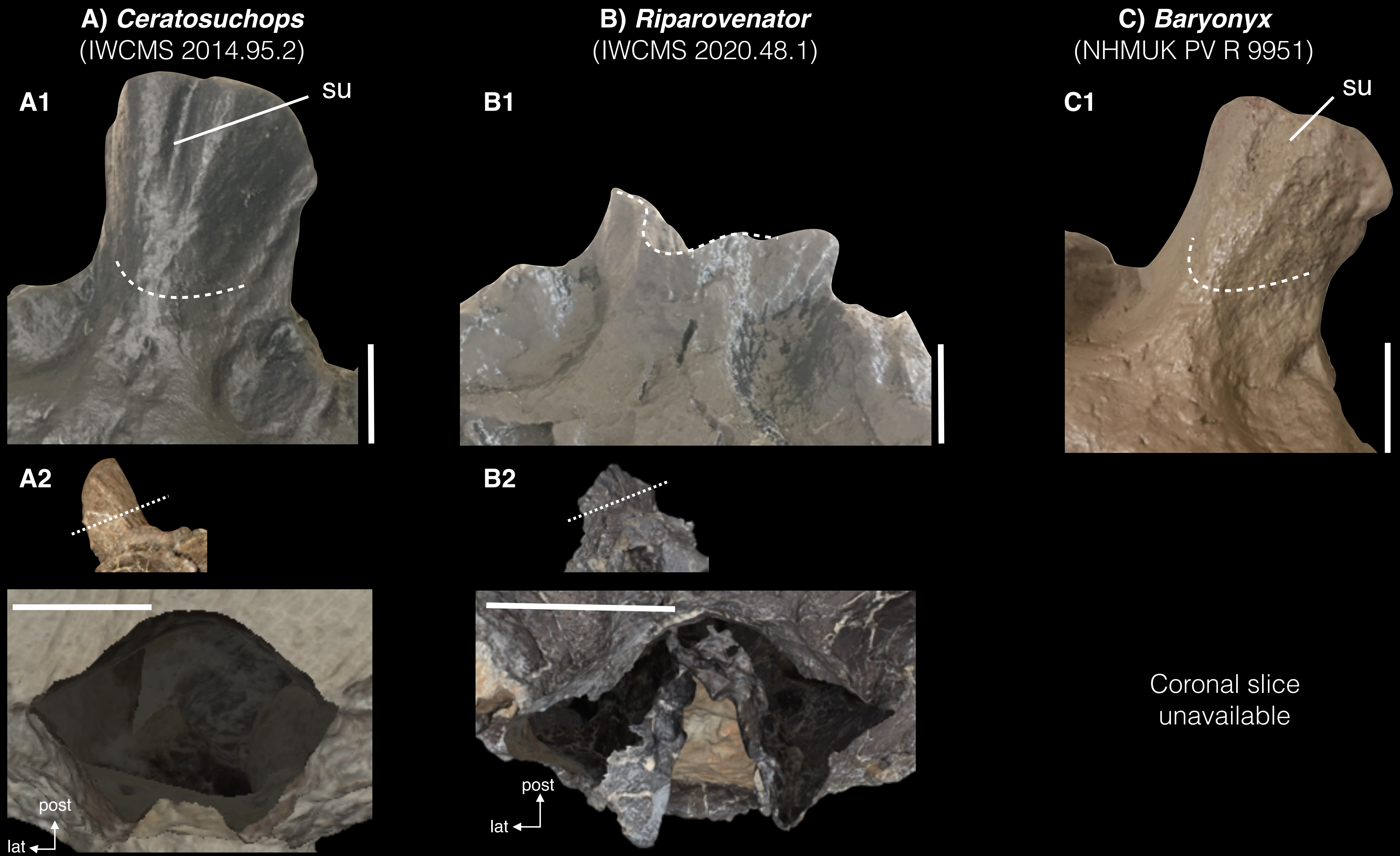

5. Supraoccipital dorsal process possessing a gently curving posteroventral surface in coronal section. A1–C1) supraoccipitals in posterior-oblique view. A2, B2) coronal slices of the dorsal process, generated in MeshLab. Note that the coronal slice of *Ceratosuchops* is based on an older surface scan of the un-prepared block, and the endocranial portion of the dorsal process is infilled with matrix. Abbreviations: su: sulcus. Scale bars: 20mm.

The posterior surface of the dorsal process in *Ceratosuchops* (below the dorsal sulcus) is gently curved (A2), and does not form the distinct and broad midline ridge of *Riparovenator*, which is clearly distinguished from the flatter lateral sides of the posterior dorsal process (B2). Whilst a coronal section was unavailable for *Baryonyx*, the ventral posterior surface of the dorsal process also differs from *Ceratosuchops*. possessing a more V-shaped cross-section.

3. Imagery of other distinguishing traits of *Ceratosuchops inferodios* relative to *Baryonyx walkeri* and/or *Riparovenator milnerae*

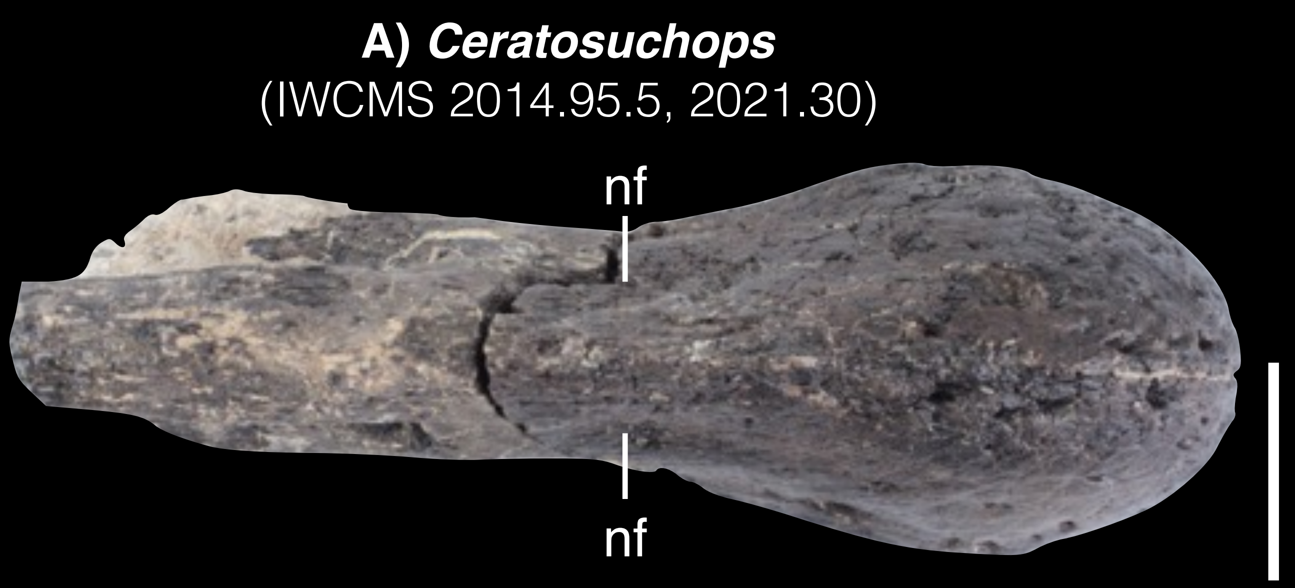

6. Presence of narial fossa. Premaxillae in dorsal view. Abbreviations: nf, narial fossa. Scale bar: 50mm.
We were unable to identify narial fossae in our first hand examination of the *Baryonyx* type specimen; as such, our observations corroborate Hendrickx et al., (2016: SI).

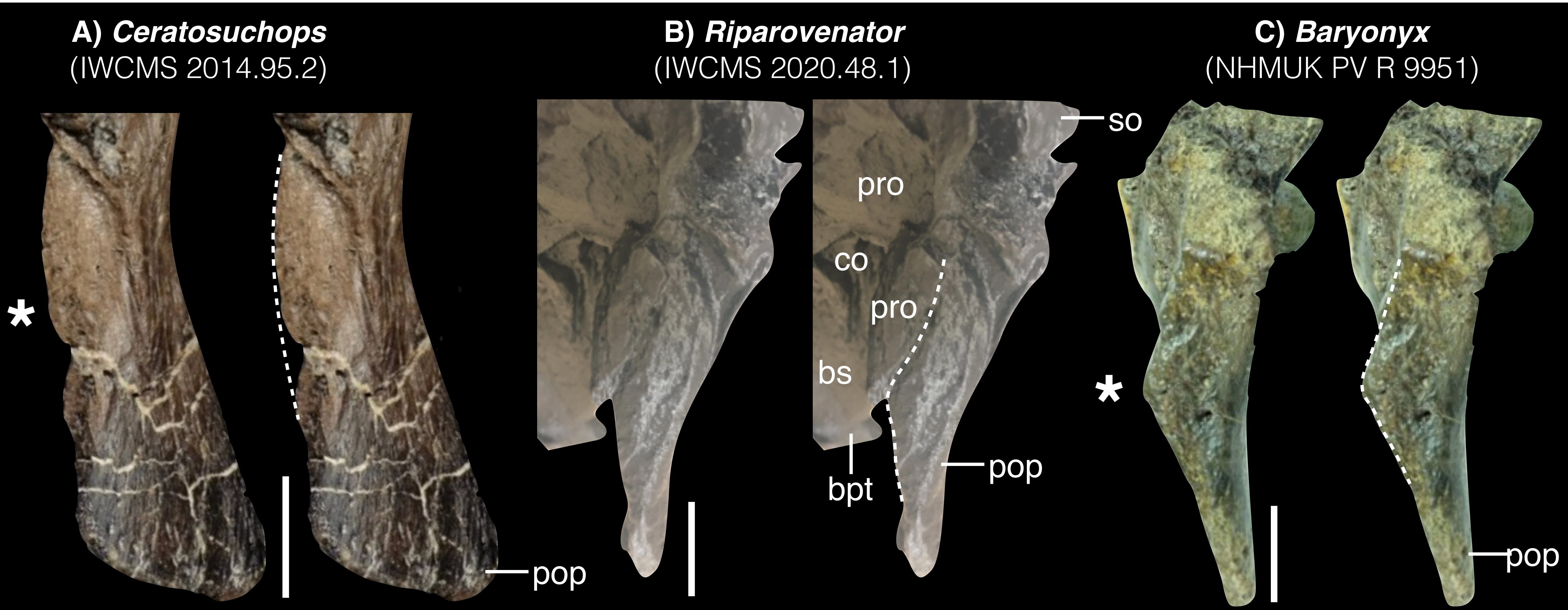

7. Gently curved anterior margin of the dorsal facet of the paroccipital process. A–C) Left paroccipital processes in dorsal view. Asterisk marks the lateral extremity of the prootic contact on the anterior surface of the process. Abbreviations: bpt, basipterygoid process; bs, basisphenoid; co, columellar recess; pop, paroccipital process; pro, prootic; so, supraoccipital. Scale bars: 20mm.

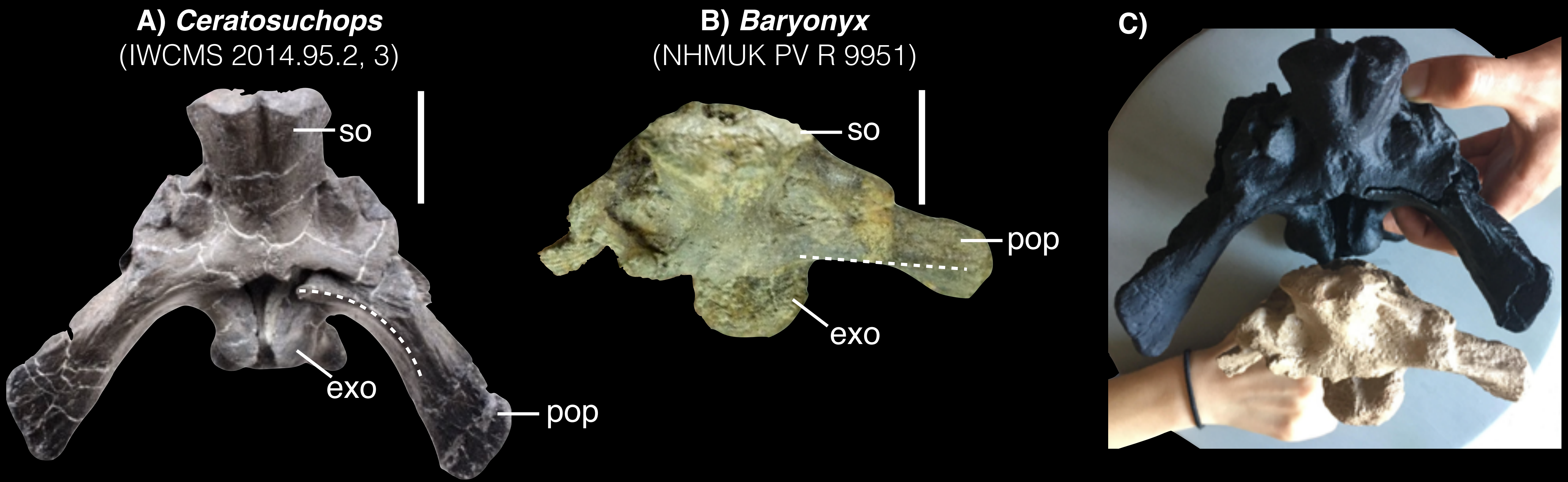

8. Posterolaterally projecting paroccipital processes. A, B) Basicranium in dorsal view. C) Comparison of life-size casts/3D prints of *Ceratosuchops* (top) and *Baryonyx* (bottom) in dorsal view. Abbreviations: exo, exoccipital; pop, paroccipital process; so, supraoccipital. Scale bar: 20mm.

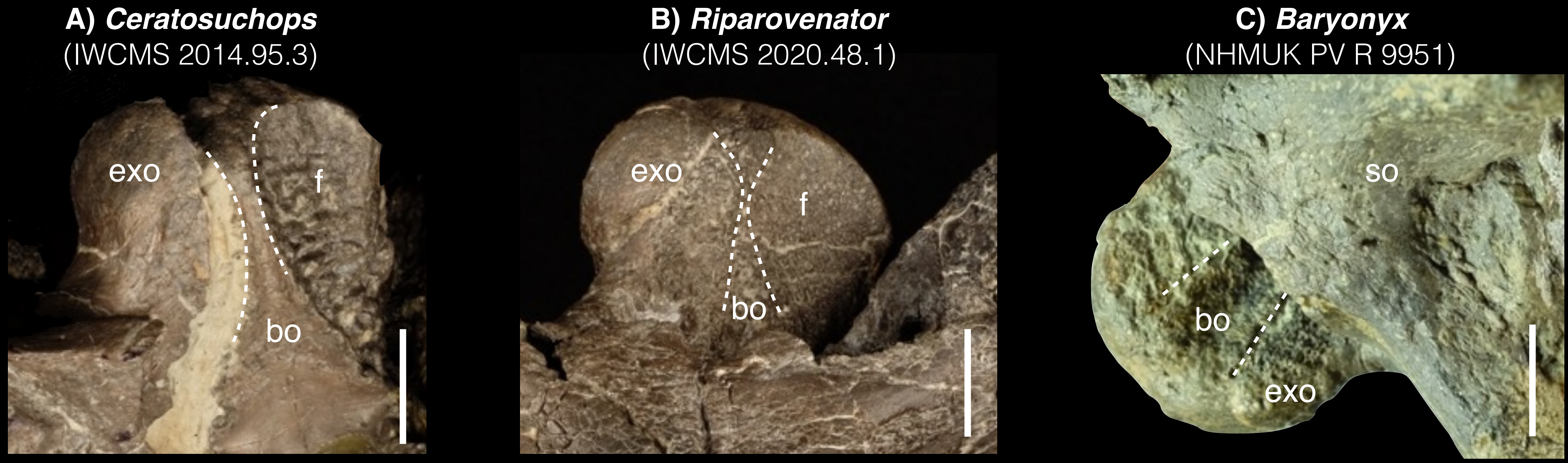

9. Closely placed exoccipitals on the dorsal occipital condyle. A–C) Occipital condyle in dorsal view. Abbreviations: bo, basioccipital; exo, exoccipital; f, exposed exoccipital facet on basioccipital; so, supraoccipital. Scale bars: 20mm.

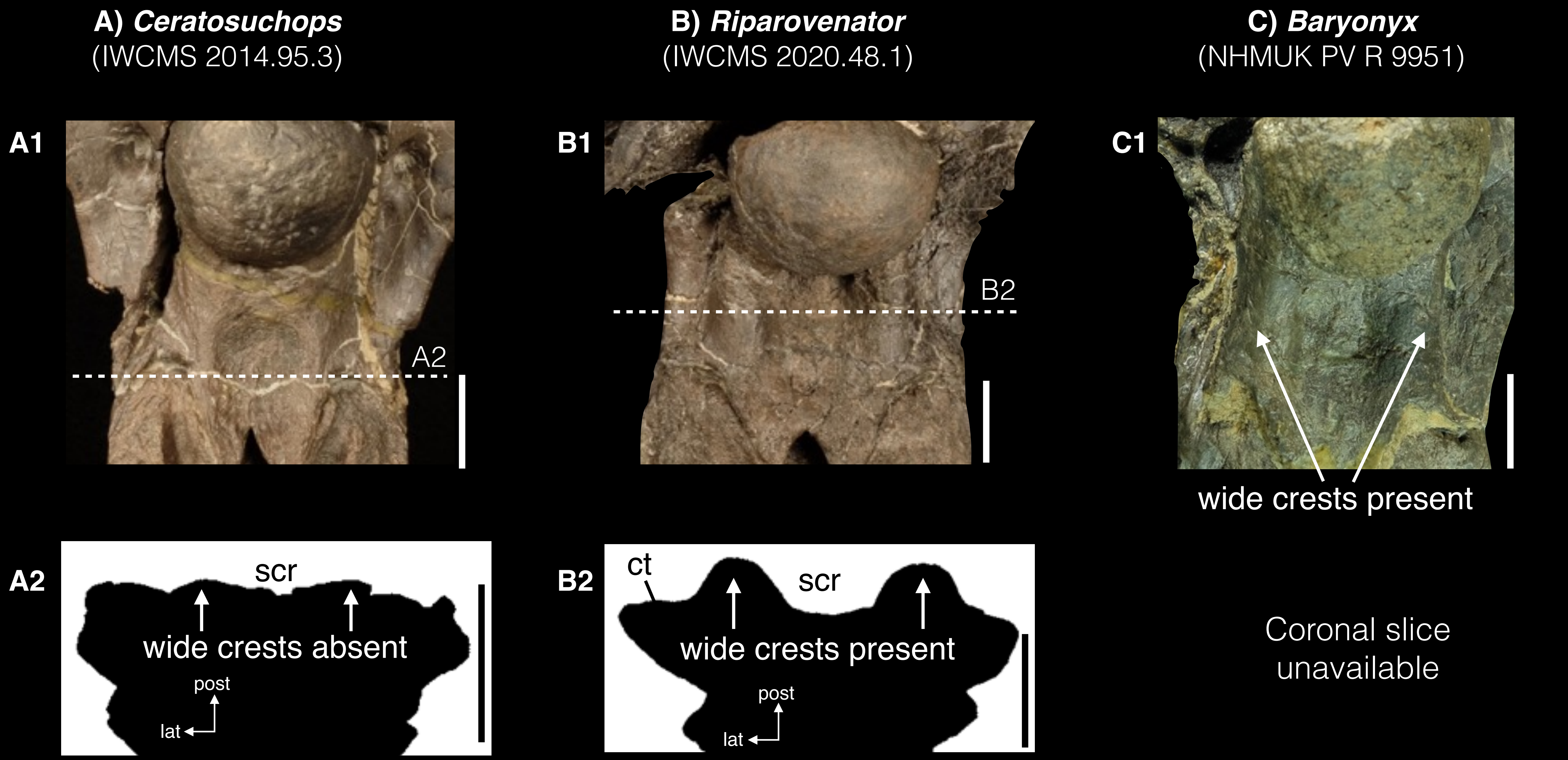

10. Reduced borders of the subcondylar recess; mediolaterally wide crests absent. A1–C1) Basioccipitals in posterior view; white line denotes coronal section. A2–B2) coronal slices through the dorsoventral mid-point of the subcondylar recess, obtained in MeshLab. Abbreviations: ct, crista tuberalis; scr: subcondylar recess.Scale bars: 20mm.

The recess in *Ceratosuchops* is barely bordered by raised lateral margins, unlike the case in *Riparovenator* and *Baryonyx*. In the latter pair, the borders are mediolaterally wide, and in the case of *Riparovenator*, notably prominent due to its diagnostically deep recess (see below).

4. Imagery of the diagnostic traits of *Riparovenator milnerae*

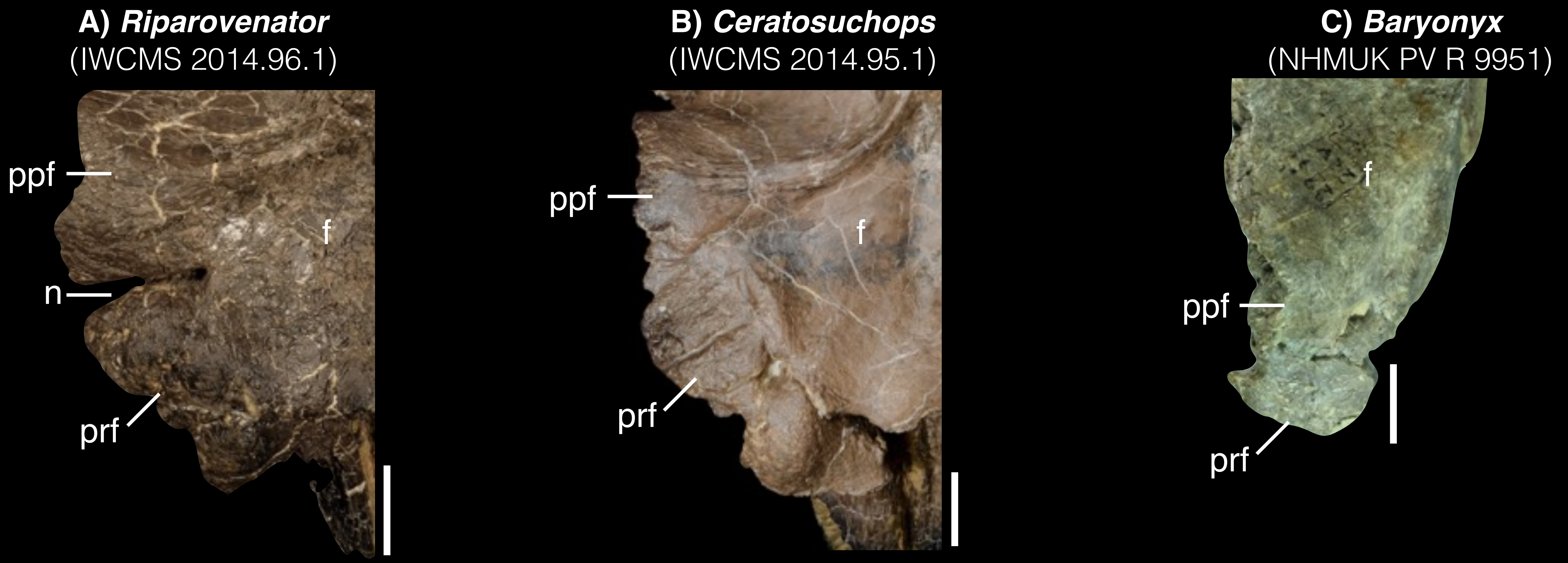

1. Presence of a notched dorsal orbital margin. A–C) Frontals in dorsal view. Abbreviations: f, frontal; n, notch; ppf, postorbital process of the frontal, prf, prefrontal. Scale bars: 20mm.

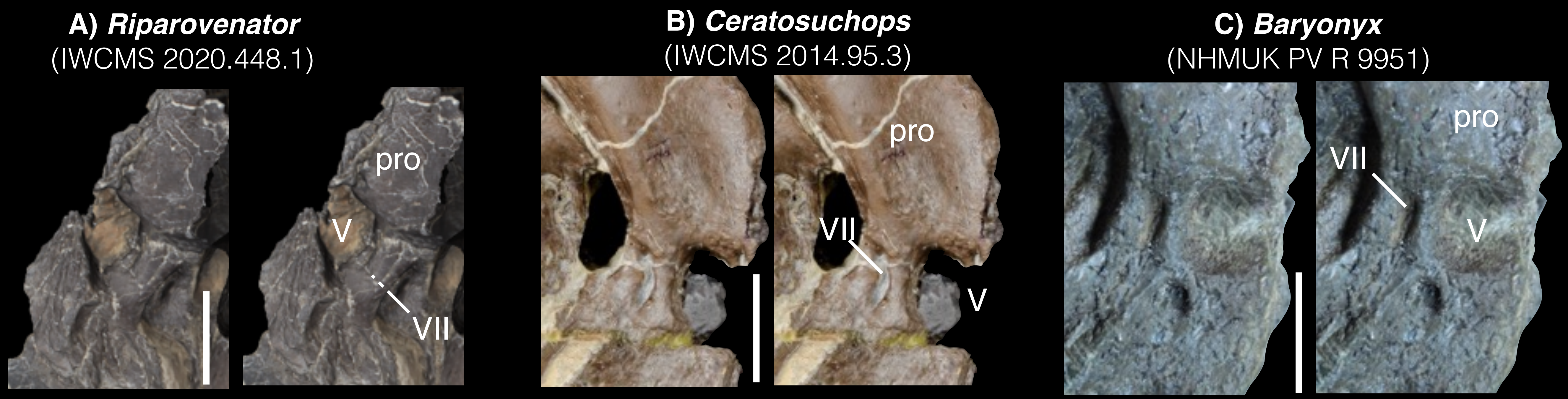

2. Deeply inset facial nerve (CN VII) foramen; foramen largely obscured in lateral view. Stereopairs of the prootics in A) left lateral view; B, C) right lateral view. Abbreviations: pro, prootic; V, trigeminal nerve foramen; VII facial nerve foramen. Scale bars: 20mm.

A) *Riparovenator*
(IWCMS 2020.448.1)

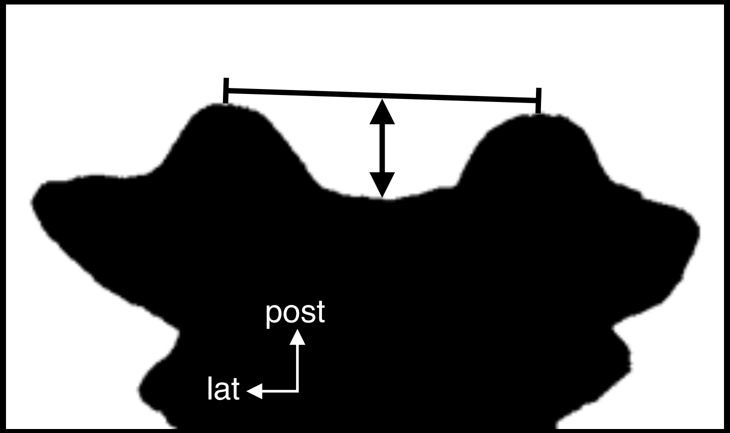

Recess depth 37% of width

B) *Ceratosuchops*
(IWCMS 2014.95.3)

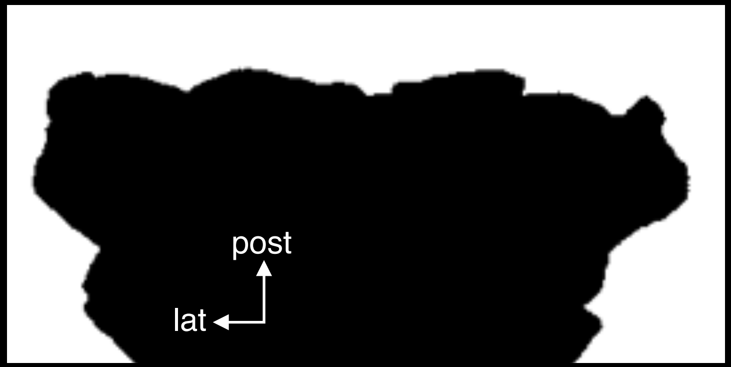

Recess depth 12% of width

C) *Baryonyx*
(NHMUK PV R 9951)

Coronal slice unavailable

Recess depth 18% of width

3. Deep subcondylar recess (over 1/3 of recess mediolateral width). A, B) coronal section of the basicranium. The schematic in A) denotes the measurements taken to calculate the relative depth of the subcondylar recess; measurements taken at the dorsoventral midpoint of the recess.

A) *Riparovenator*
(IWCMS 2020.448.1)

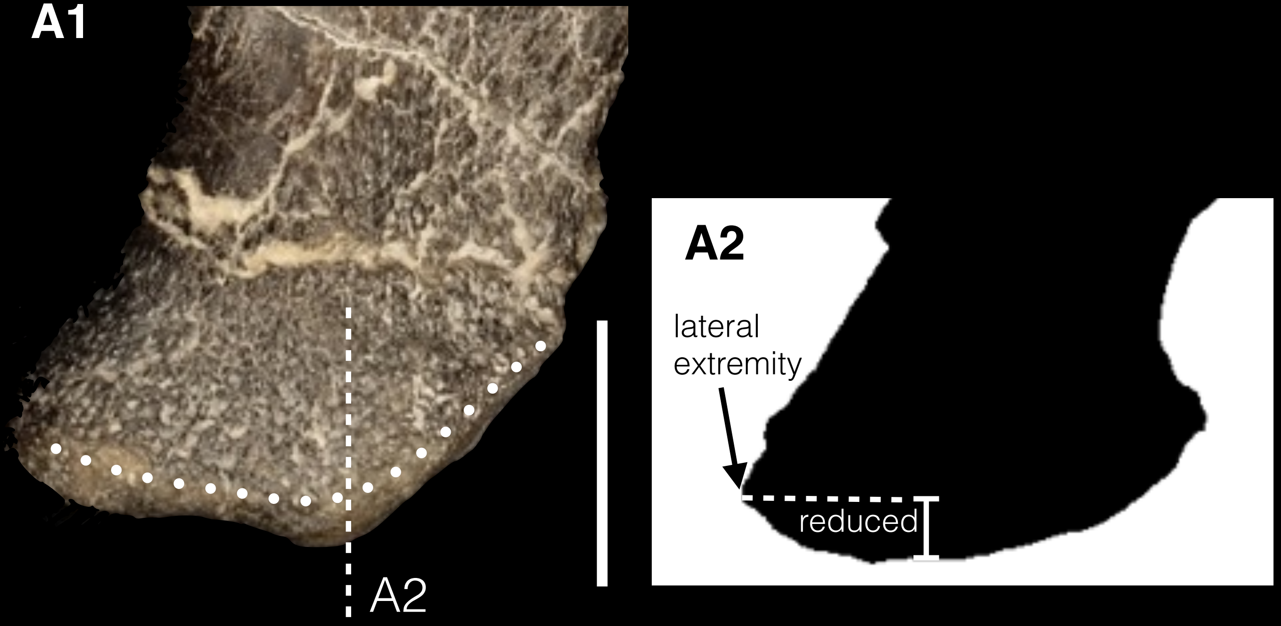

B) *Ceratosuchops*
(IWCMS 2014.95.3)

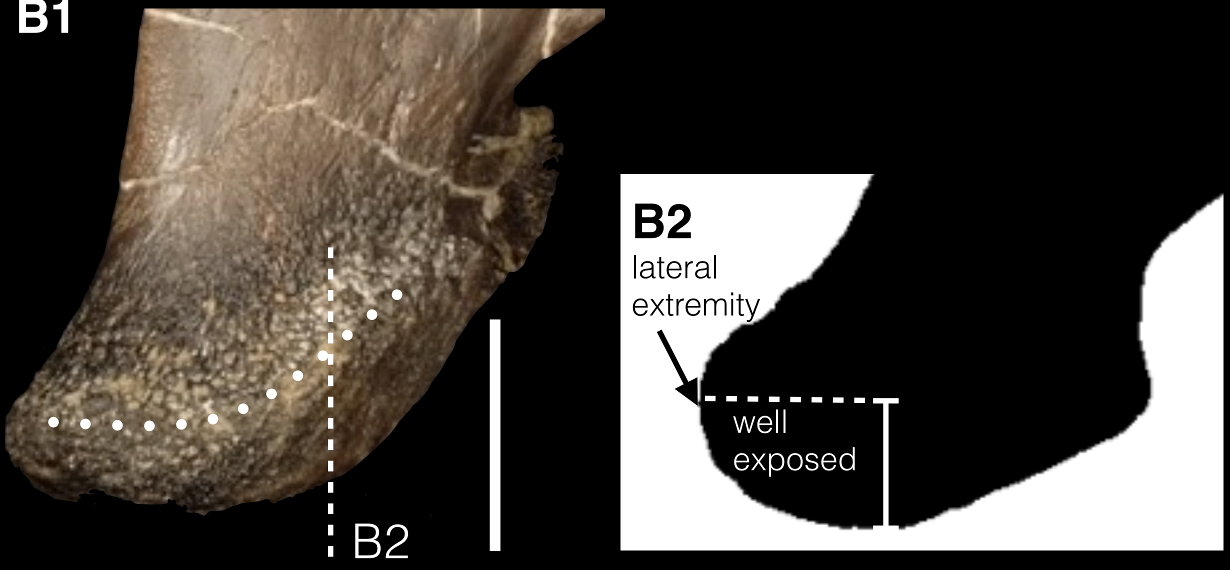

4. Reduced exposure of the ventral surface of the basipterygoid processes in lateral view. A1, B1) Right basipterygoid processes in lateral view; A2, B2) transverse slices of of the basipterygoid processes (scaled to the same mediolateral width). Dotted lines in A1 and B1 denote the lateral extremity of the processes; the vertical dashed lines represent the transverse slice pictured in A2 and B2. Scale bars: 20mm.

5. Imagery of other distinguishing traits of *Riparovenator milnerae* relative to *Baryonyx walkeri* and/or *Ceratosuchops inferodios*

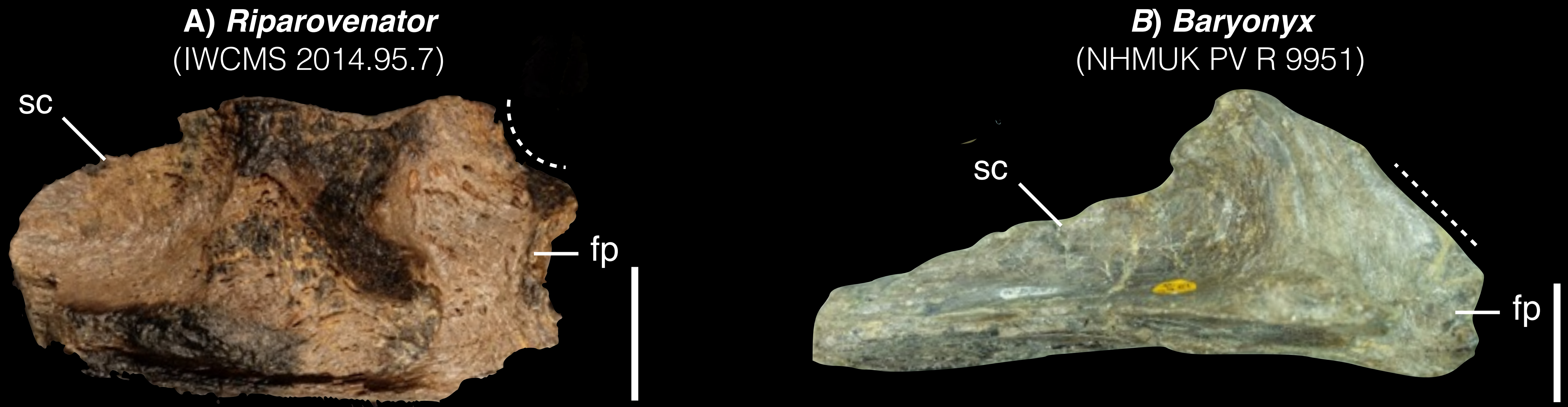

5. Curved dorsal margin of the frontal process of the nasal. A, B) posterior nasal fragments in left lateral view. Abbreviations: fp, frontal process; sc, sagittal crest. Scale bars: 20mm.

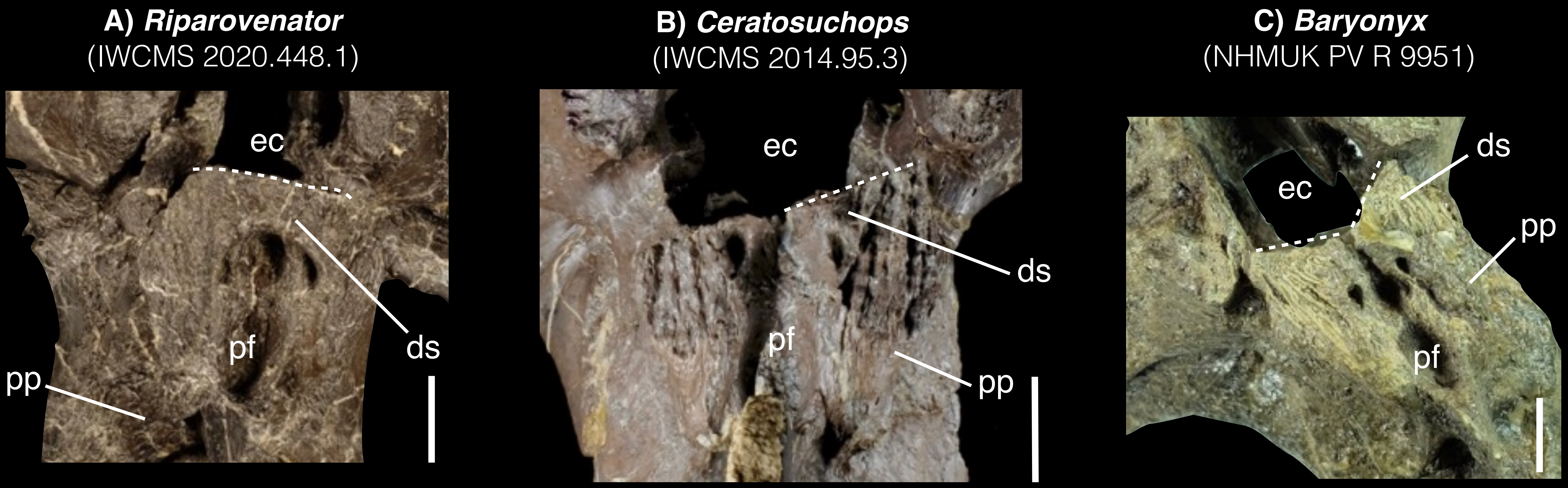

6. Straight dorsal margin of the dorsum sellae. A–C) basicrania in anterior view. Note that the right hand portion of the dorsum sellae has been lost in *Ceratosuchops*, however mirroring the complete left portion indicates that the dorsal margin would assume a shallow V-shape, as in *Baryonyx*. Abbreviations: ds, dorsum sellae; ec, endocranial cavity; pp, preotic pendant; pf, pituitary fossa. Scale bars: 20mm.

7. Closely placed exoccipitals on the dorsal occipital condyle. See *Ceratosuchops*, trait 9.

---

8. Prominent borders of the subcondylar recess. See *Ceratosuchops*, trait 10.

A) *Riparovenator* (IWCMS 2020.448.1) **B) *Ceratosuchops*** (IWCMS 2014.95.3) **C) *Baryonyx*** (NHMUK PV R 9951)

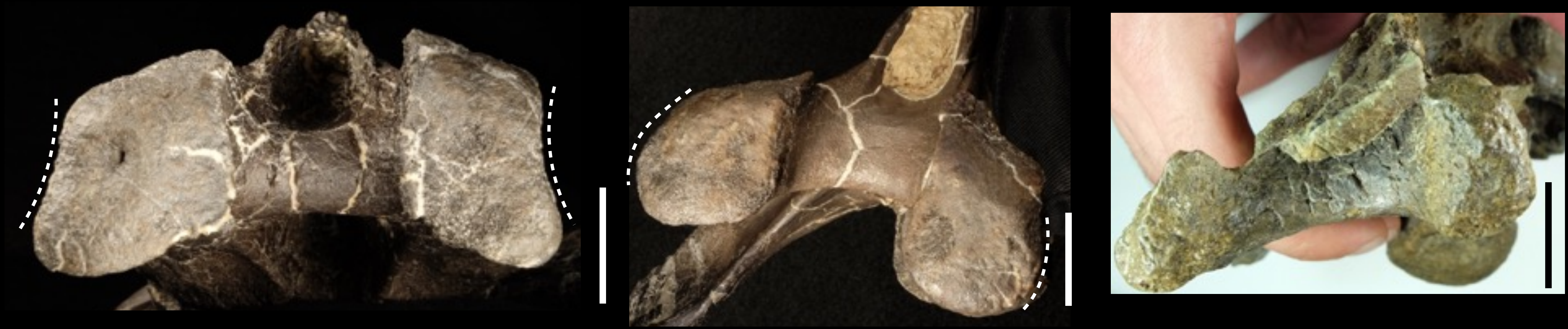

9. Lateral margins of the basipterygoid processes concave in ventral view. A–C) basipterygoid process in ventral view. Note that the processes are too damaged in *Baryonyx* to provide comparison. Scale bars: 20mm.
